# Ceria-Zirconia nanoparticles reduce intracellular globotriaosylceramide accumulation and attenuate kidney injury by enhancing the autophagy flux in cellular and animal models of Fabry disease

**DOI:** 10.1186/s12951-022-01318-8

**Published:** 2022-03-09

**Authors:** Jong Hun An, Sang-Eun Hong, Seong-Lan Yu, Jaeku Kang, Chang Gyo Park, Hoi Young Lee, Sung-Ki Lee, Dong Chul Lee, Hwan-Woo Park, Won-Min Hwang, Sung-Ro Yun, Yohan Park, Moon Hyang Park, Kuk Ro Yoon, Se-Hee Yoon

**Affiliations:** 1 Division of Nephrology and Department of Internal Medicine, Myunggok Medical Research Institute, College of Medicine, Konyang University, Daejeon, Republic of Korea; 2 Department of Chemistry, Hannam University, Daejeon, Republic of Korea; 3 Myunggok Medical Research Institute, College of Medicine, Konyang University, Daejeon, Republic of Korea; 4 Department of Pharmacology, College of Medicine, Konyang University, Daejeon, Republic of Korea; 5 Department of Obstetrics and Gynecology, College of Medicine, Konyang University, Daejeon, Republic of Korea; 6 Personalized Genomic Medicine Research Center, Korea Research Institute of Bioscience and Biotechnology (KRIBB),, Deajeon, Republic of Korea; 7 Department of Cell Biology, Myunggok Medical Research Institute, Konyang University College of Medicine, Daejeon, Republic of Korea; 8 Department of Pathology, College of Medicine, Konyang University, Daejeon, Republic of Korea

**Keywords:** Ceria-Zirconia nanoparticle, Fabry disease, Autophagy flux, Globotriaosylceramide

## Abstract

**Background:**

Fabry disease (FD) is a lysosome storage disease (LSD) characterized by significantly reduced intracellular autophagy function. This contributes to the progression of intracellular pathologic signaling and can lead to organ injury. Phospholipid–polyethyleneglycol-capped Ceria-Zirconia antioxidant nanoparticles (PEG-CZNPs) have been reported to enhance autophagy flux. We analyzed whether they suppress globotriaosylceramide (Gb3) accumulation by enhancing autophagy flux and thereby attenuate kidney injury in both cellular and animal models of FD.

**Results:**

Gb3 was significantly increased in cultured human renal proximal tubular epithelial cells (HK-2) and human podocytes following the siRNA silencing of α galactosidase A (α-GLA). PEG-CZNPs effectively reduced the intracellular accumulation of Gb3 in both cell models of FD and improved both intracellular inflammation and apoptosis in the HK-2 cell model of FD. Moreover these particles attenuated pro fibrotic cytokines in the human podocyte model of FD. This effect was revealed through an improvement of the intracellular autophagy flux function and a reduction in reactive oxygen species (ROS). An FD animal model was generated in which 4-week-old male *B6;129-Gla*^*tm1Kul*^/J mice were treated for 8 weeks with 10 mg/kg of PEG-CZNPs (twice weekly via intraperitoneal injection). Gb3 levels were reduced in the kidney tissues of these animals, and their podocyte characteristics and autophagy flux functions were preserved.

**Conclusions:**

PEG-CZNPs alleviate FD associated kidney injury by enhancing autophagy function and thus provide a foundation for the development of new drugs to treat of storage disease.

**Graphical Abstract:**

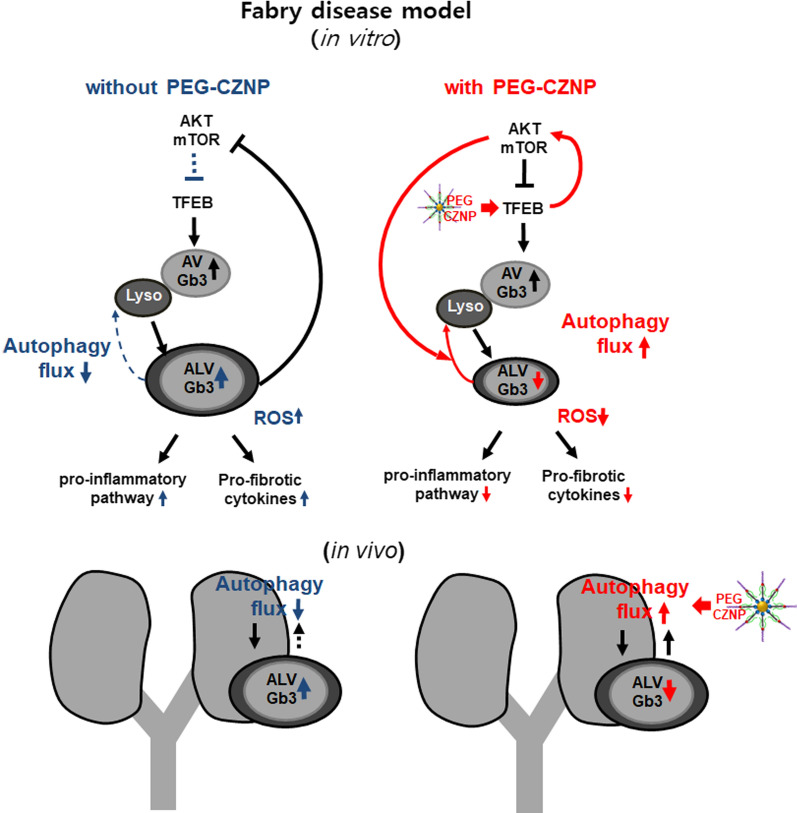

**Supplementary Information:**

The online version contains supplementary material available at 10.1186/s12951-022-01318-8.

## Introduction

Fabry disease (FD) is a lysosome storage disorder (LSD) caused by a deficiency, deletion, or disrupted function of the α-galactosidase A (α-GLA) enzyme, and is X chromosome related [1]. The prevalence of FD among the male population is reported to range from 1 in 40,000 to 60,000 [2, 3], but these figures are likely to be an underestimation. The accumulation of intracellular ceramide trihexoside, called as globotriaosylceramide (Gb3), in FD-affected individuals over time causes irreversible progressive damage to various tissues and major organs such as the kidneys, heart, and nerves and ultimately results in early death [4]. Enzyme replacement therapy (ERT) with α-GLA supplementation is the basic treatment approach for FD [5]. However, the long term benefits of ERT remain to be determined in terms of reducing the risk of morbidity and mortality related to FD [6]. In addition, a lifelong intravenous ERT regimen is burdensome and there can be issues with the onset of resistance to these therapies caused by the formation of antibodies to α-GLA [7]. A pharmacological chaperone, migalastat (1-deoxygalactonojirimycin), which is administered oral, has recently been released, but is effective only for patients with specific genetic mutations [8, 9]. New therapeutic approaches to overcome the limitations of current FD treatments are being trialed, including second generation ERTs [10], gene recombination therapies using DNA and viral vectors [11–14], and substrate reduction therapies [15, 16], but remain challenging from an efficacy and safety perspective. Autophagy lysosomal pathway dysfunction is a key pathogenic event in LSD [17–20] and it has been reported that intracellular autophagy function is significantly disrupted in these disorders, including FD [18, 21, 22]. The intracellular accumulation of Gb3 is caused by an α-GLA deficiency but the combination of this loss of enzyme function with autophagy perturbation can enhance this accumulation and thereby further promote intracellular pathologic signaling. Although the restoration of an impaired autophagy flux in FD is postulated to alleviate disease progression, only a few studies to date have described the manipulation of the autophagic pathway as a strategy for treating LSD [23–25].

Some nanoparticles (NPs) are known to affect autophagy, either as activators [26–28] or inhibitors [29, 30] of this process, depending on their type. Ceria nanoparticles (CNPs) were found previously to activate the lysosome-autophagy system and enhance autophagic clearance [28]. When zirconia is attached to CNPs (CZNPs), the ceria atom tends to remain in its Ce^3+^ form which enhances the efficacy of the nanoparticle as a free radical scavenger mimicking superoxide dismutase (SOD) activity, and produces a more stable efficacy compared to CNPs [31, 32]. Due to hydrophobic nature of synthesized CZNPs, capping CZNPs with polyethylene glycol (PEG) improve the NPs water dispensability [31, 33]. Indeed, the autophagy flux restoring ability of polyethylene glycol-capped CZNPs (PEG-CZNPs) was reported in our previous study [32].

We speculated that PEG-CZNPs would effectively alleviate the progression of FD by promoting Gb3 metabolism through their antioxidant and autophagy flux enhancer properties. We evaluated the changes of intracellular Gb3 accumulation and signaling in cellular and animal models of FD and confirmed the efficacy of PEG-CZNPs in alleviating the progression of kidney damage associated with FD.

## Material and methods

### Materials

Cerium(III) acetylacetonate hydrate, zirconium(IV) acetylacetonate (97%), oleylamine (technical grade, 70%), N-Acetyl-L-cysteine (NAC, A7250), FITC (fluorescein isothiocyanate, isomer I (suitable for protein labeling, ≥ 90% (HPLC), powder) were purchased from Sigma Aldrich (St. Louis, MO). 18:0 PEG2000 PE (1,2-distearoyl-sn-glycero-3-phosphoethanolamine-N-[methoxy(polyethylene glycol)-2000] (ammonium salt, powder), and DSPE-PEG(2000) amine (1,2-distearoyl-sn-glycero-3-phosphoethanolamine-N-[amino(polyethylene glycol)-2000] (ammonium salt, powder) were purchased from Avanti® Polar Lipids, Inc. (Alabaster, AL). Acetone (99.5%, extra pure), and chloroform (99.5%, extra pure) were obtained from SAMCHUN PURE CHEMICALS (Seoul, Korea). Deionized water (DW) was produced using HIQ-II® (CORETECH, KOREA).

### Synthesis of phospholipid–polyethylene glycol-capped Ceria-Zirconia nanoparticles

PEG capped CZNPs were synthesized using a non-hydrolytic sol–gel reaction method as described previously [32]. We also used the PEGylation method to make water-dispensable CZNPs [31, 33]. The synthesis process of PEG-CZNPs is briefly described in Additional file 1: Supplementary method and schematically shown in Additional file 1: Fig.S1A. The PEG-CZNPs were then characterized using field-emission transmission electron microscopy (FE-TEM), selected area electron diffraction (SAED), energy dispersive X-ray spectroscopy (EDS) (Tecnai G^2^ S-TWIN; FEI Company, Eindhoven), bio-high voltage electron microscope (Bio-HVEM, JEM-1400 Plus and JEM-1000 BEF; JEOL Ltd.,Tokyo), multi-purpose X ray-diffractometer (XRD, X’Pert Powder; Malvern Panalytical, Malvern), thermogravimetric analysis (TGA, SCINCO TGA 1000; Scinco Co., Seoul), dynamic light scattering measurements (DLS, Zetasizer Nano-ZS system; Malvern Instruments Ltd., Malvern). 1,1-diphenyl-2-picrylhydrazyl (DPPH) free radical scavenging assays were performed to examine the antioxidant capacity of PEG-CZNPs as described previously [32]. To confirm the intracellular localization of PEG-CZNPs we have made FITC conjugated PEG-CZNPs. The process of the preparation of FITC conjugated PEG-CZNPs is briefly shown in in Additional file 1: Supplementary method and Fig. S1B,C.

### Cell culture

HK-2 cells (a human renal proximal tubular epithelial cell line) were purchased from the Korean cell line bank (KCLB®, Seoul**,** South Korea) and cultured in RPMI-1640 medium supplemented with 10% fetal bovine serum (FBS) and 1% penicillin/streptomycin at 37℃ under 5% CO_2_ in a humidified incubator.

Conditionally human immortalized podocytes (AB8/13 cell line) were generously provided by Professor Moin Saleem (University of Bristol, Southmead Hospital, Bristol, UK) [34]. These cells were grown in RPMI-1640 supplemented with 10% FBS, 1% penicillin/streptomycin and 1% insulin-transferrin-selenium supplement (ITS-G; Gibco, Grand Island, NY). The cells were propagated at 33℃ until reaching 80% confluence and were allowed to differentiate at 38℃ with 40–50% confluence for 14 days. Differentiation was assessed by immunofluorescence (IF) staining with podocin (Sigma Aldrich; St. Louis, MO), nephrin (Abcam, Cambridge, MA) and synaptopodin (Santa Cruz Biotechnology, Santa Cruz, CA) using a confocal laser-scanning microscope (LSM710; Carl Zeiss, Jena, Germany) (Additional file 1: Fig. S2A, B).

### RNA interference

Small interfering RNA (siRNA) targeting *α-GLA* was purchased from OriGene Technologies (Rockville, MD, ORG-SR320297). HK-2 cells and human mature podocytes were reverse transfected with this siRNA using Lipofectamine RNAiMAX transfection reagent (Invitrogen, catalog number: 13778150) in Opti-MEN medium (Thermo Fisher Scientific, Gibco™, catalog number: 31985070). The medium was changed at 6 h post-transfection to RPMI supplemented with 10% FBS. To overcome the low efficiency and short blocking times of siRNA interference, we performed a second transfection with the *α-GLA* siRNA at 4 days after the first transfection in HK-2 cells. These *α-GLA* knockdown HK-2 cells were harvested 8 days after the first transfection for analysis of Gb3, and assays for ROS and, cell viability. In human mature podocytes, which do not proliferate after maturation, *α-GLA* siRNA was performed once only, and these cells were harvested at 5 days after transfection. The silencing efficacy of the siRNA was examined to assess whether it could fully suppress the expression and activity of GLA in both cultured HK-2 cells and human podocytes (Additional file 1: Fig. S3A, B).

For the transcription factor EB (TFEB) translocation assay, cells were transfected with pEGFP-N1-TFEB (Addgene, 38119, deposited by Shawn Ferguson) using Lipofectamine 3000 (Invitrogen, Carlsbad, CA) in accordance with the manufacturer’s protocol. Transfection of pEGFP-TFEB was performed together with the second transfection of *α-GLA* siRNA in the HK-2 cells at four days later after the first *α-GLA* siRNA transfection. pEGRP-TFEB was also co-transfected with *α-GLA* siRNA in the human matured podocytes.

### Cell exposure to PEG-CZNPs

Toxicity tests were performed at various concentration of PEG-CZNPs by 3-(4,5-dimethylthiazole-2-yl)-2,5-diphenyltetrazolium bromide (MTT) assay in HK-2 cells and mature podocytes. The process of MTT assay is briefly shown in Additional file 1: Supplementary method. Among them 10 ug/mL, an effective concentration with no cellular toxicity, was selected (Additional file 1: Fig. S4). HK-2 cells were continuously exposed to PEG-CZNPs at a concentration of 10 μg/mL for 8 days from the day of the first *α-GLA* siRNA transfection and the medium containing these particles was changed once per day. Human mature podocytes were exposed to the PEG-CZNPs once at a concentration of 10 μg/mL for the first 24 h from the day of the *α-GLA* siRNA transfection, and cells were harvested after 5 days.

### Quantitative real-time RT-PCR

Total RNA was extracted using Trizol (Invitrogen, Carlsbad, CA), and 1 μg samples were then reverse transcribed as described previously [35–37]. It was performed using an iQ SYBR Green Supermix (Bio-Rad Laboratories, Hercules, CA) The following primer sets were then used for real-time PCR analysis of the indicated genes: *P62/SQSTM*, 5’GCACCCCAATGTGATCTGC-3’ (forward) and 5’CGCTACACAAGTCGTAGTCTGG-3’ (reverse); *TGF β*, 5’CAACAATTCCTGGCCATACCT-3’ (forward) and 5’CAACCACTGCCGCACAACTCC-3’ (reverse); *Fibronectin*, 5’GCGAGAGTGCCCCTACTACA-3’ (forward) and 5’GTTGGTGAATCGCAGGTCA-3’ (reverse); *α-smooth muscle chain (SMA)*, 5’CGCACAACTGGCATCGTGCTGGAC-3’ (forward) and 5’TGATGTCCCGGACAATCTCACGCT-3’ (reverse); *Collagen IV*, 5’GGAGTACCAGGACAAGCTCAA-3’ (forward) and 5’CACCTTTTTGGCCCTTTTCTC-3’ (reverse); *Caspase 4*, 5’CTCTGAGGCTCTTTCCAACG-3’ (forward) and 5’TTCCAACACCTTAAGTGGCTTT-3’ (reverse); *MCP-1*, 5’CCCCAGTCACCTGCTGTTAT-3’ (forward) and 5’AGATCTCCTTGGCCACAATG-3’ (reverse); *HES-1*, 5’CCAAAGACAGCATCTGAGCA -3’ (forward) and 5’TCAGCTGGCTCAGACTTTCA-3’ (reverse); *GAPDH*, 5’GTCGGAGTCAACGGAT -3’ (forward) and 5’AAGCTTCCCCGTTCTCAG-3’ (reverse);.

Data were normalized to *GAPDH* as an endogenous control. Relative expression differences were calculated using the 2^−(ΔΔCt)^ method.

### Immunoblotting

Immunoblotting was performed as described in our previous studies [35–37]. Antibodies raised against the following proteins were used in the analyses: p-p38, and p- c-Jun N-terminal kinase (p-JNK) (Cell signaling Technology; Denver, MA); microtubule-associated proteins 1A/1B light chain 3B (LC3B) (Sigma Aldrich; St. Louis, MO); GLA (Abcam, Cambridge, MA); p38, JNK, extracellular signal-regulated kinase (ERK), p-ERK, and β-actin (Santa Cruz Biotechnology, Santa Cruz, CA).

### Reactive oxygen species measurements

Dihydroethidium (DHE; Thermo Fisher Scientific, Rockford, IL), 2′,7′-dichlorodihydrofluorescein diacetate (DCF-DA; Sigma Aldrich, St. Louis, MO), and dihydrorhodamine (DHR) 123 (Sigma Aldrich) were used to measure the cellular ROS levels in accordance with the manufacturer’s recommendations and as described in our previous studies [35–37].

### Immunofluorescence assay (IFA)

For analysis by IFA, HK-2 cells and human podocytes were first incubated on coverslips in 6-well plates at a density of 1 × 10^4^/ml and 3 × 10^3^ /ml, respectively. The cells were then rinsed with PBS and fixed for 15 min at room temperature with 4% paraformaldehyde. This was followed by further rinsing with PBS and permeabilization with 0.3% Triton X-100 in PBS. Non-specific binding was blocked with 1% bovine serum albumin (BSA) for 1 h at room temperature prior to incubation with primary antibodies overnight at 4℃ (prepared in 1% BSA). The primary antibodies used in theses analyses were raised against Gb3 (TCI, A2506, 1:250), LC3B (Sigma, P0372, 1:250), phospho- mammalian target of rapamycin (p-mTOR; CST, 5536 s, 1:250), p-protein kinase B (p-AKT; CST, 4508L, 1:250), mTOR (CST, 2983 s, 1:250), AKT (SantaCruz, SC5298, 1:250), synaptopodin (Santa Cruz, SC-515842, 1:250), podocin (Sigma, L7543, 1:500), and nephrin (Abcam, ab267351, 1:250). After further washing the next day with PBS, the coverslips were incubated for 1 h at room temperature with Cy2 or Cy3 conjugated anti-rabbit (Jackson Immunoresearch Laboratories, Cy2 111–225-144; Cy3, 111-165-144; 1:1000) or anti-mouse (Jackson Immunoresearch Laboratories, Cy2 115-225-003; Cy3, 115-165-003; 1:1000) secondary antibodies in 1% BSA. The nuclei were counterstained with 6-diamidino-2-phenylindole (DAPI; Molecular Probes, Carlsbad, CA) for 15 min and the stained slides were mounted in anti-fade-fluorescence mounting medium (DAKO, S3023). For the GRP-TFEB translocation assay pEGFP-TFEB transfected cells were seeded in 6-well plates and incubated for 4 days (HK-2 cells) or 5 days (matured human podocytes). The cells were treated as indicated in the text, washed, and fixed. Images were analyzed using confocal microscopy (LSM710; Carl Zeiss, Jena, Germany).

### Analysis of PEG-CZNP cellular uptake and intracellular localization using fluorescence

To investigate the cellular uptake of PEG-CZNPs, FITC-conjugated PEG-CZNPs were administered to mature human podocytes. At 4, 8, 16, 24, 48, 72 and 96 h after this incubation at 38℃ in 5% CO_2_ in a dark room, the cells were washed twice with PBS, and fixed with 2.5% paraformaldehyde (PFA). The nuclei were counterstained with DAPI, and cells were observed under a confocal laser-scanning microscope (LSM710; Carl Zeiss, Jena, Germany). The intracellular location of the PEG-CZNPs was confirmed using Mitotracker (Mitotracker Orange CMTMRos; Thermo Fisher Scientific, Carlsbad, CA) and Lysotracker (Lyso Tracker Blue DND-22. Thermo Fisher Scientific, Inc.) reagents. The uptake and accumulation of PEG-CZNPs in human podocytes was also examined with a HT7700 electron microscope (Hitachi Ltd., Tokyo, Japan) at a 7500–50,000 × magnification. Images were recorded using a AMT XR81-B CCD camera (AMT, Woburn, MA).

### Gb3 isolation from human podocytes

Intracellular Gb3 quantification in human podocytes was conducted by SCL (Seoul Clinical Laboratories, Seoul) via Liquid Chromatography coupled to Electrospray Ionization Tandem Mass Spectrometry (LC–ESI–MS/MS). Briefly, approximately 10^7^ human podocytes were homogenized in 100 μl of water using ultrasonic VCX 750 device (SONICS, Newtown, CT). The total protein content of the preparation was then routinely determined using bicinchoninic acid (BCA). After the addition of 500 μl of methanol, 250 μl of chloroform and 5 μg/ml of an internal standard, N-C17:0-ceramide trihexoside (Matreya, State College, PA) were also added. Lipids were extracted overnight at 48℃. Interfering glycerolipids were degraded by alkaline hydrolysis with 75 μl of 1 M potassium hydroxide in methanol for 2 h at 37℃. After neutralization with glacial acetic acid, the samples were vortexed for 30 s and centrifuged at 12,000 g for 5 min at 4℃. The upper layer was evaporated under nitrogen gas and the dried lipid extracts were resolved in 200 μl of the LC mobile phase solvent, and sonicated for 5 min. After further centrifugation (12,000 g, 5 min, 4℃), 100 μl of the clear supernatant was transferred to an auto-injector vial. LC-MA/MS analysis was performed using a reverse phase guard column (C8L, 4 × 3 mm ID; Pehnomenex, Aschaffenburg, Germany) with detection using an API4000 triple quadrupole mass spectrometer (SCIEX, Toronto, Canada). The LC (1200 Binary LC System; Agilent, Waldbronn, Germany) was operated with a gradient elution at a flow rate of 350 μl/min and a mobile phase of 0.1% formic acid and 2 mM ammonium acetate in methanol; 5 μl of each sample was injected. The total run time was 5 min. Gb3 species were monitored in the positive ion mode using their specific multiple reaction monitoring (MRM) transitions. The Gb3 quantifier peaks were integrated using the Analyst 1.4.2 software (AB SCIEX, Framingham, MA).

### Flow cytometry analysis of apoptotic HK-2 cells

HK-2 cells were stained with a combination of Annexin V-FITC, propidium iodide (PI), and analyzed by flow cytometry analysis (CytoFLEX, Beckman Coulter; Indianapolis, IN). Annexin V positive HK-2 cells were monitored using an EzWay Annexin V-FITC apoptosis detection kit (catalog number K29100; KOMA Biotech, Seoul, Korea) in accordance with the manufacturer’s protocol and as previous study [38]. Briefly, HK-2 cells subjected to *GLA* knockdown by using two siRNA transfections were harvested, rinsed three times with PBS, and incubated in 1 × Binding Buffer. The cells were then incubated with 1.25 μL of Annexin V-FITC and 10 μl of propidium iodide (PI) at room temperature for 15 min in the dark. The samples were analyzed using a flow cytometry analysis (CytoFLEX; Beckman Coulter, Indianapolis, IN). The percentage of apoptosis was calculated as the number of PI and Annexin V positive cells divided by the total number of cells. The experiments were repeated tree times independently.

### Animal model of FD

FD model mice, *B6;129-Gla*^*tm1Kul*^/J (also known as α-Gal A KO mice) [39], were purchased from the Jackson Laboratory (JAX stock #003535, Bar Harbor, ME) and were maintained by brother X sister mating. The male KO mice used in this study were selected by PCR-based genotyping using ear punch DNA samples. The primers used to amplify the α-GalA gene were as follows: oIMR5947 5'-AGGTCCACAGCAAAGGATTG-3'; oIMR5948 5'-GCAAGTTGCCCTCTGACTTC-3'; and oIMR7415 5'-GCCAGAGGCCACTTGTGTAG-3' [40]. The mice were raised in groups of 2–3 per cage under standard housing conditions and with a standard rodent diet in a pathogen-free animal facility. The experimental groups of these animals were as follows (all male): Group1 (wild type, normal saline injection; n = 3), Group 2 (wild type + PEG-CZNP injection n = 3), Group 3 (*B6;129-Gla*^*tm1Kul*^/J mice + normal saline injection; n = 5) and Group 4 (*B6;129-Gla*^*tm1Kul*^/J mice + PEG-CZNP injection; n = 5). Group 1 and 3 were administered 2 mL/kg of normal saline intraperitoneally twice per week from 4 to 12 weeks of age. Groups 2 and 4 received 10 mg/kg (2 mL/kg) infection of PEG-CZNPs instead of normal saline. The scheme for this animal study is shown in Additional file 1: Fig. S5.

All animal procedures used in this study were reviewed and approved by the Institutional Animal Care and Use Committee(IACUC) of Konyang Research Institute and were conducted in accordance with guidelines established by the Association for Assessment and Accreditation of Laboratory Animal Care International (AAALAC International).

### α-galactosidase (α-GLA) activity assay

α-GLA activity in mouse blood samples was determined using a commercial assay kit (Cat #K407-100; Biovision, Milpitas, CA) according to the manufacturer’s protocol and previously reported methods [41]. Briefly, the mouse plasma samples were mixed with 2 mM 4-methylum-bellifery-a-D-galactopyranoside (or 4MU-α-Gal) and 50 mM n-Acetyl Galactosamine in 10 mM Sodium Citrate Buffer (pH 4.6). The reaction mixture was then incubated in 37℃ for 30 min and terminated by the addition of glycine-Na-OH (500 Mm) buffer (pH 10.5). α-GLA activity was expressed as nmol/h/mL plasma.

### Biochemical and histological analysis of the mouse FD model

Blood samples were taken for biochemical analysis, and kidney tissues were harvested to assess for any pathological changes from each separate group of mice at 12 weeks of age. The serum blood urea nitrogen (BUN) and creatinine levels were measured using a Fugi Dri Chem 3500 device (Fugifilm, Tokyo, Japan). Kidney tissues were fixed in 10% phosphate buffered formalin. After subsequent embedding in paraffin, these sections were cut at a 4 μm thickness and stained with hematoxylin–eosin (H&E) and Periodic Acid Schiff (PAS) to observe any structural changes. IFA was also performed Using primary antibodies against Gb3 (Amsbio, Cambridge, MA), synaptopodin (Sigma Aldrich) and LC3B (Sigma Aldrich). Electron microscopy was also conducted. Briefly, the mouse kidney tissues were fixed overnight with 2.5% glutaraldehyde in 0.1 M phosphate buffer (pH 7.4) at 4℃, and post-fixed in 1% OsO_4_ in 0.1 M phosphate buffer for 1 h. After dehydration in graded ethanol, the samples were embedded in Epok-812 (Ohken shohi; Chuo-ku, Tokyo, Japan). Ultrathin Sects. (90 nm) were then prepared using an ultra-microtome (Ultracult-N; Reichert Nissei, Heidelberg, Germany), stained with uranyl acetate and lead citrate, and micrographed with a TEM (Hitachi H-600A; Hitachi Ltd., Tokyo, Japan). To examine the biodistribution of PEG-CZNP, we have measured ceria and zirconia contents in the organs of B6 mice after intraperitoneal injection of 10 mg/kg PEG-CZNPs twice a week for 12 weeks using inductively coupled plasma-mass spectrometry (ICP-MS, ICAP TQ, Thermo Scientific, Darmstadt, Germany). Further details of this method are available as Additional file 1: Supplementary method.

### Statistical analysis

All graphed data are expressed as a mean ± standard deviation and were statistically analyzed using the Student’s t-test. Differences were considered significant for *P*-values of < 0.05.

## Results

### Characteristics of the PEG-CZNPs

We successfully synthesized under 3 nm PEG-CZNPs. The characteristics of these particles were evaluated using FE-TEM, scanning transmission electron microscopy (STEM), SAED, EDS, XRD, TGA, DLS and Zeta potential analysis. To confirm CZNP structural stability over time, the crystal structures and chemical composition of CZNP were measured by XRD and SAED at 12 week interval. There was no change in composition between the NPs at the two time points. We also investigated the size distribution using DLS immediately after NPs synthesis and after 12 weeks. The size distribution of PEG-CZNP was 14.55 ± 2.86 nm when measured immediately after synthesis and 13.43 ± 2.09 nm after 12 weeks. The mean size distribution of NPs examined at the 12-week intervals overlapped within the standard deviation (SD). The antioxidant capacity of the CNP, CZNP and PEG-CZNP was measured using a DPPH assay. At a concentration of 10 μg/mL, PEG-CZNPs showed superior antioxidant properties comparable to CNPs or CZNPs through the scavenging of free radicals. We also utilized N-Acetyl-L-cysteine (NAC), a well-known antioxidant, as a positive control and compared its antioxidant potency with that of PEG-CZNPs. PEG-CZNPs showed antioxidant activity comparable to that of 0.5 mM of NAC. (Fg. 1A-I).

**Fig. 1:**
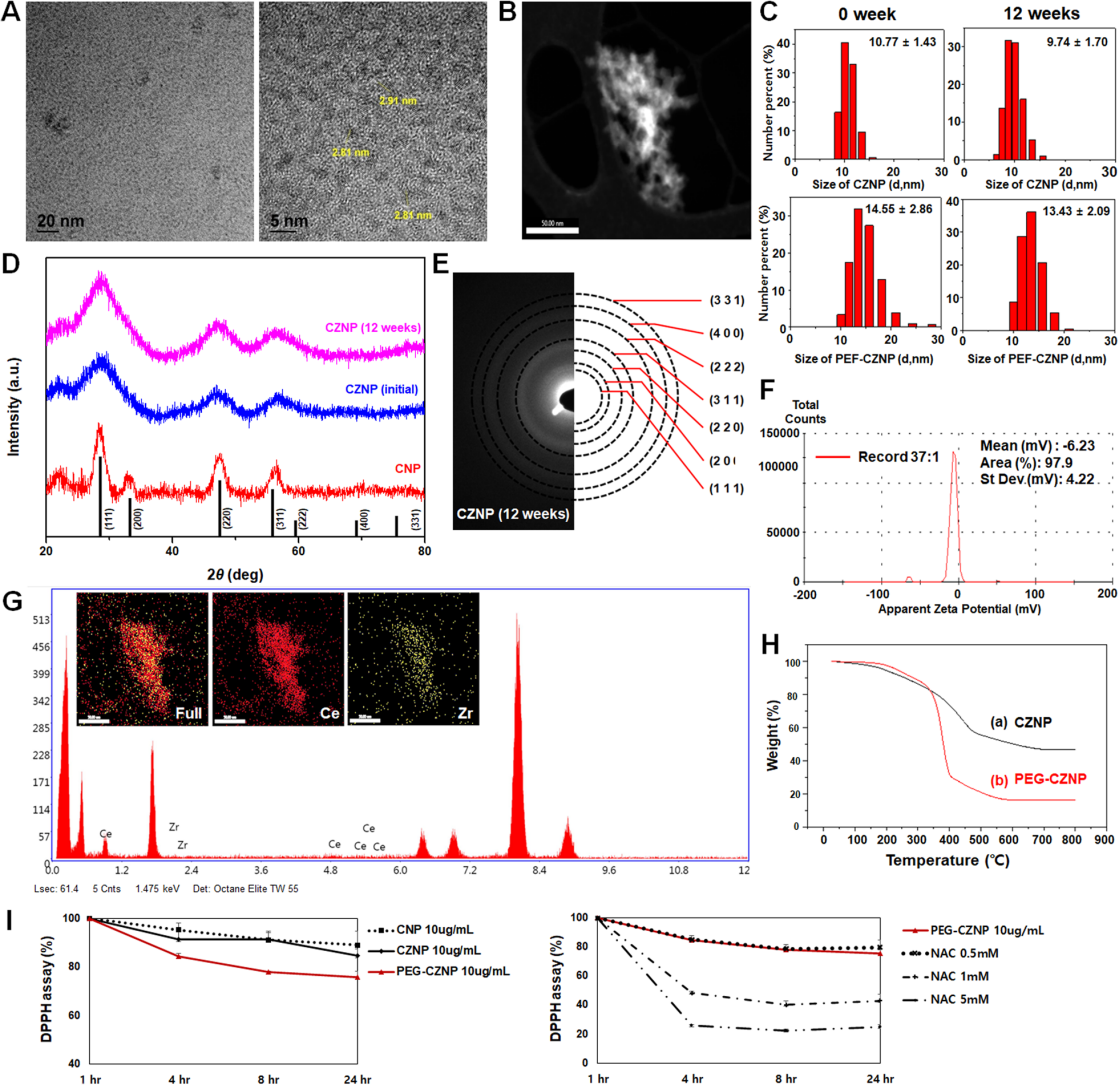
Characteristics of the PEG-CZNPs. **A**, **B** High-resolution transmission electron microscopy images (TEM) and low-magnification scanning transmission electron microscopy (STEM) images revealing the highly crystalline nature of the PEG-CZNPs. **C** Particle size distribution of CZNPs and PEG-CZNPs in deionized water, measured by dynamic light scattering (DLS). **D** Comparison of the X-ray diffraction (XRD) spectra for CNPs, and CZNPs at 0 and 12 weeks. The black lines represent the ceria reference peaks. **E** Corresponding selected area electron diffraction (SAED) patterns of CZNPs at 12 weeks. **F** Zeta potential curve obtained from PEG-CZNPs (Zeta potential, mV; -7.33). **G** Use of energy dispersive X-ray spectroscopy (EDS) spectra of the CZNPs to confirm their atomic compositions; 67 at% Ce and 33 at% Zr. **H** Thermogravimetric analysis (TGA) curves of CZNPs and PEG-CZNPs. **I** Antioxidant effects of the PEG-CZNPs assayed using 2,2-diphenyl-a-picrylhydrazyl (DPPH) at a concentration of 10 μg/mL over 4, 8, and 24 h. Data were analyzed with the Student’s t test (mean ± SD)

### Intracellular localization and biodistribution of the PEG-CZNPs

**Fig. 2:**
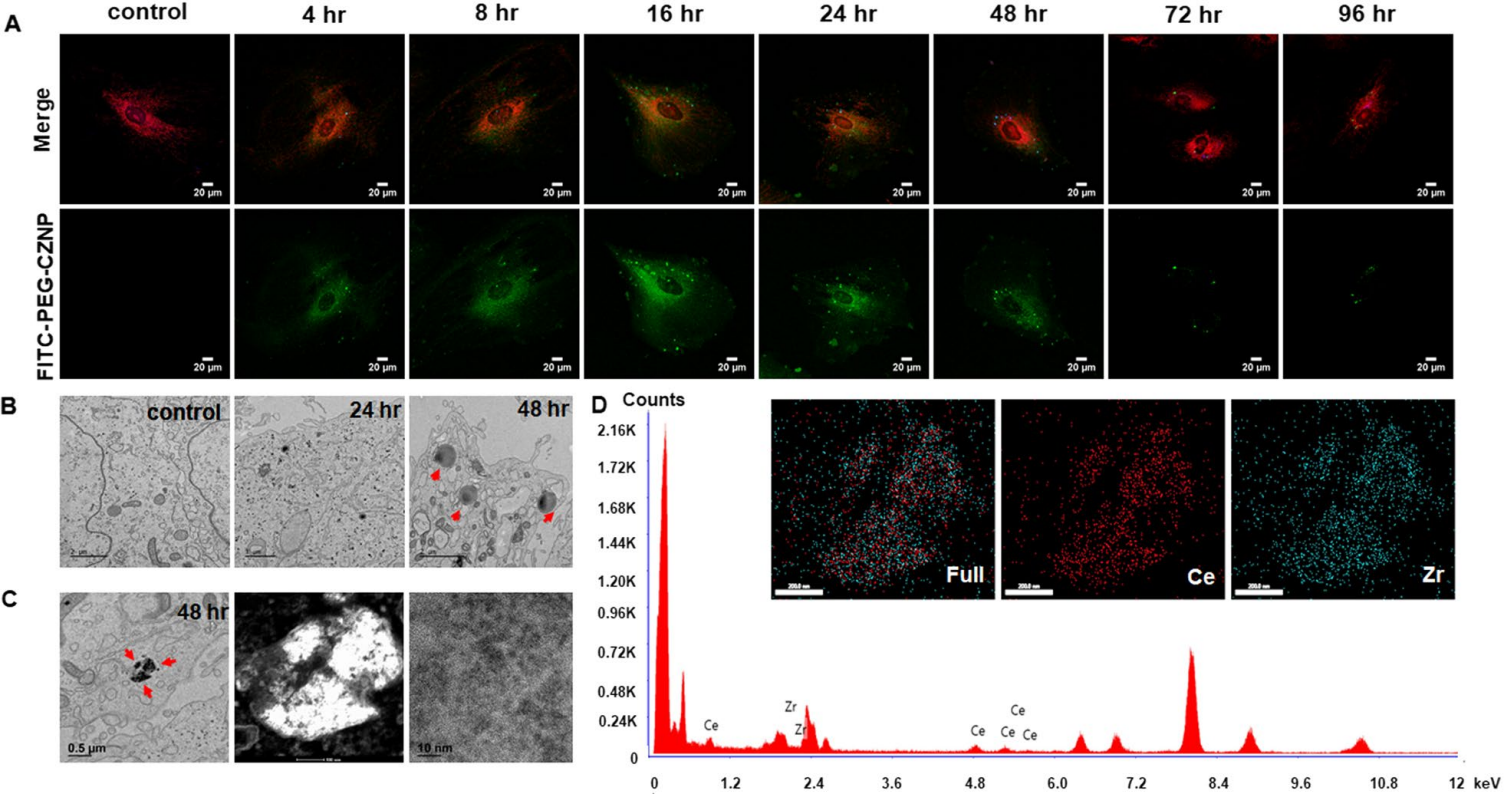
Intracellular localization and biodistribution of the PEG-CZNPs. **A** Confocal microscopy analysis of human podocyte treated with FITC-labeled PEG-CZNPs after 0, 4, 8, 16, 24, 48, 72 and 96 h. The human podocytes were then double-stained with Mitotracker (orange) and Lysotracker (blue). **B** TEM images of human podocytes treated with PEG-CZNPs after 0, 24, and 48 h. Residual PEG-CZNPs were found to be distributed in the lysosomes (red arrow). **C** STEM images of PEG-CZNPs in the lysosome of human podocytes **D** EDS spectra of PEG-CZNPs in the lysosomes of human podocytes to confirm their atomic compositions; 67 at% Ce and 32 at% Zr

We confirmed the intracellular localization of the PEG-CZNPs in HK-2 cells in previous studies [32]. Thereby, we newly analyzed the intracellular localization of these particles in mature human podocytes. FITC-labeled PEG-CZNPs were administered to mature human podocytes to investigate their intracellular localization and observed by confocal microscopy at 1, 4, 8, 16, 24, 48, 72 and 96 h after exposure (Fig. 2A). The cytoplasmic levels of PEG-CZNPs increased up to 16 h and decreased gradually thereafter. At the 72 h timepoints, these particles showed a localization that matched that of the lysosomes (Additional file 1: Fig. S6). In electron microscopy (EM) revealed that the lysosome sizes had markedly increased at 48 h after exposure to the PEG-CZNPs and that black particles were visible inside them (Fig. 2B). We then analyzed the particles found in the lysosomes using STEM and EDS and observed that they showed the characteristics of PEG-CZNPs (Fig. 2C, D). In biodistribution analysis, ceria and zirconia accumulated the most in the spleen followed by the liver. However, there was a little accumulation in the lung and kidneys and they were rarely observed in the brain (Additional file 1: Fig. S7).

### PEG-CZNPs suppress intracellular Gb3 accumulation in both HK-2 cells and the human podocytes model of FD

We evaluated the changes of Gb3 levels in HK-2 cells and human podocytes after *GLA* knockdown with or without PEG-CZNP treatment. A strong Gb3 expression level was observed in the *GLA* knockdown HK-2 cells and human podocytes by IF and EM imagery. Notably, the intracellular Gb3 accumulation level was significantly suppressed by the PEG-CZNP treatments. To confirm this observation in the human podocyte model of FD, we quantified the Gb3 levels by LC–MS/MS and again detected a significant decrease in the total intracellular Gb3 content after PEG-CZNP treatment (Fg. 3A–I).

**Fig. 3:**
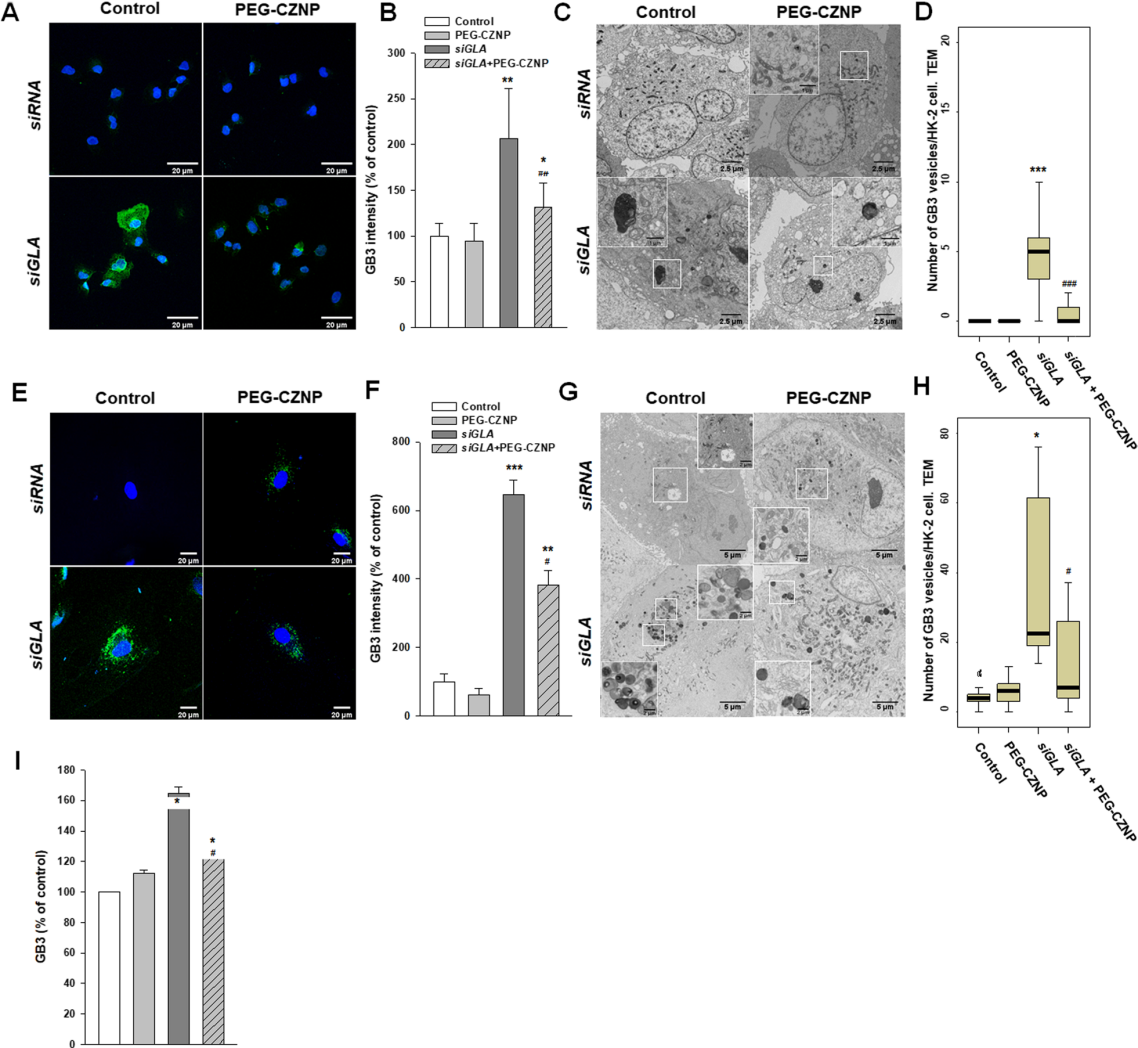
Gb3 detection in *α-GLA* knockdown HK-2 cells and human podocytes at baseline and under PEG-CZNPs treatment. **A–D** HK-2 cell analysis **A** Representative confocal immunofluorescence microscopy images of Gb3 (green), with DAPI counterstaining (blue), in control (siRNA) and α-GLA deficient HK-2 cells (siGLA), with or without exposure to PEG-CZNPs. **B** Quantification of the fluorescence intensities in the confocal microscope images using ImageJ software. **C** Representative TEM images of control (siRNA) and α-GLA deficient HK-2 cells (siGLA), with or without exposure to PEG-CZNPs. **D** Quantitative analysis of Gb3 vesicles in control (siRNA) and α-GLA deficient HK-2 cells (siGLA), with or without exposure to PEG-CZNPs. **E–I** Human podocyte analysis **E** Representative confocal immunofluorescence microscopy images of Gb3 (green), with DAPI counterstaining (blue), in control (siRNA) and α-GLA deficient human podocytes (siGLA) with or without exposure to PEG-CZNPs. **F** Quantification of the fluorescence intensity of confocal microscope images using ImageJ software. **G** Representative TEM images of control (siRNA) and α-GLA deficient human podocytes (siGLA) with or without exposure to PEG-CZNPs. **H** Quantitative analysis of Gb3 vesicles from TEM images. **I** Quantification of the total Gb3 content using by LC–MS/MS data and the Student’s t test. Data shown are the mean ± SD; *P < 0.05, **P < 0.01, and ***P < 0.001 versus control; ^#^P < 0.05, ^##^P < 0.01, and ^###^P < 0.001 versus *siGLA* knockdown alone

### PEG-CZNPs enhance the autophagy flux in both HK-2 cells and human podocytes following a *GLA* knockdown

**Fig. 4:**
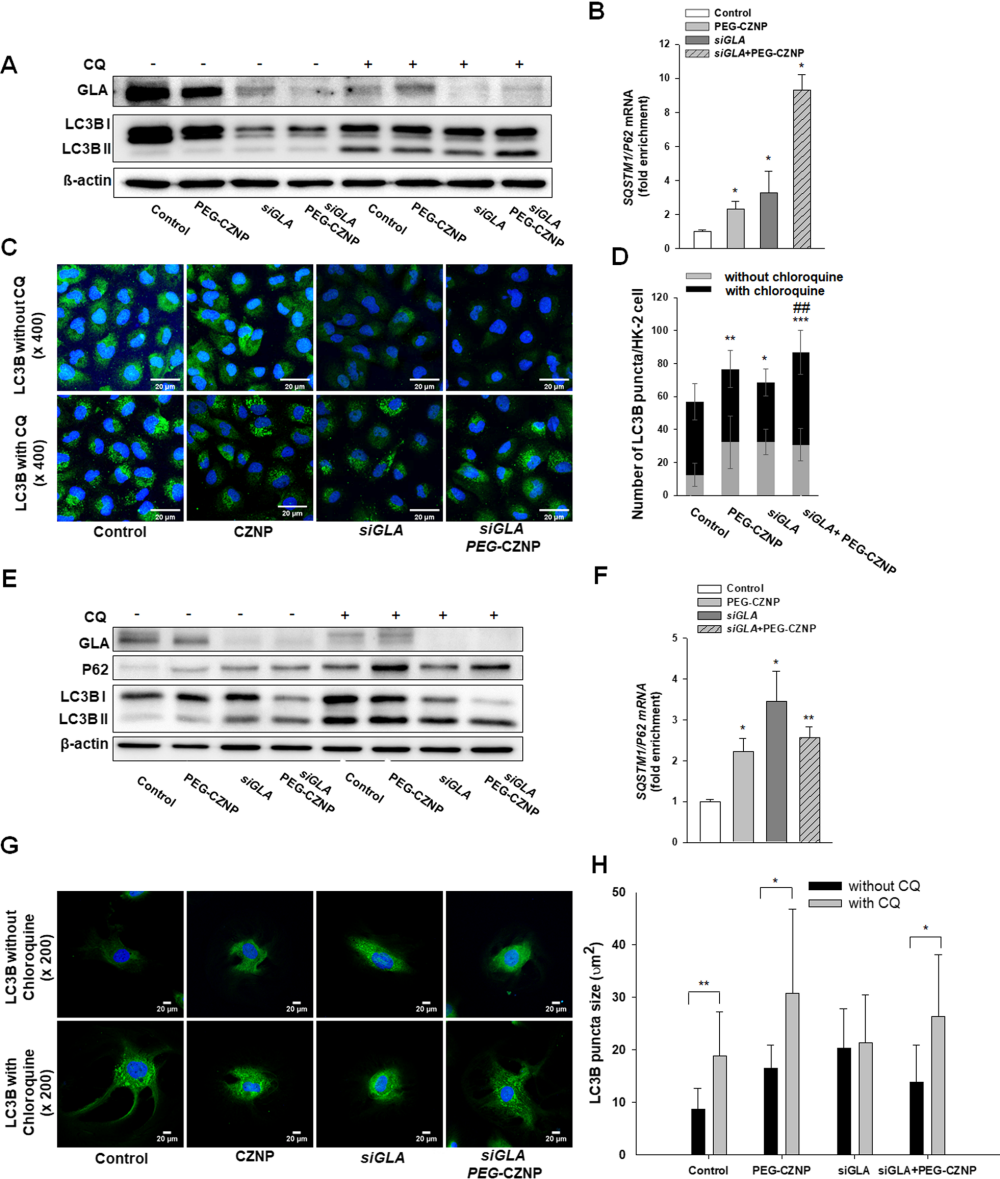
Effects of PEG-CZNPs on the autophagy response in HK-2 cells and in the human podocyte model of FD. **A-D** HK-2 cell analysis **A** Immunoblotting analysis of the autophagy response with and without chloroquine (CQ). **B** Quantitative real-time RT-PCR analysis of *SQSTM1/P62* expression. **C** and** D** Confocal microscopy images of cells stained with LC3, with or without CQ exposure, and quantification of the fluorescence intensities using ImageJ software. **E–H** Human podocyte analysis **E** Immunoblotting analysis of the autophagy response with and without CQ exposure. **F** Quantitative real-time RT-PCR analysis of *SQSTM1/P62* expression. **G** and** H** Confocal microscopy images of cells stained with LC3, with or without CQ exposure, and quantification of the fluorescence intensities using ImageJ software. Data values represent the mean ± SD; *P < 0.05, **P < 0.01, and ***P < 0.001 versus control; ^###^P < 0.001 versus *siGLA* knockdown alone

We assessed the changes in the autophagy response in both HK-2 cells and human podocytes after a *GLA* knockdown following exposure to PEG-CZNPs. This analysis was done using PCR, immunoblotting, and IFA. The *p62*mRNA expression levels increased after PEG-CZNP exposure, with or without a *GLA* knockdown, in both cell models of FD. This indicated that PEG-CZNP activates autophagy. The LC3B II/LC3B I ratio increased after the *GLA* knockdown in both cell models, as determined by immunoblotting analysis. For the evaluation of autophagy flux function, we administrated chloroquine (CQ), an autophagy inhibitor. In subsequent immunoblotting analysis, the CQ treated cells were found to have a larger increase in the LC3BII/LC3BI ratio if also exposed to PEG-CZNPs in both cell models of FD. In the IF analysis, the number of LC3B puncta in the HK-2 cells was significantly increased by CQ administration and PEG-CZNP treatment, either with or without a *GLA* knockdown. Moreover, the sizes of these LC3B puncta were significantly increased by CQ administration in all of the cells tested except the *GLA* knockdown human podocytes. These results indicated that the impaired autophagy flux in cellular models of FD is restored by PEG-CZNP treatment (Fig. 4A–H).

### PEG-CZNPs enhance TFEB nuclear translocation and contribute to the homeostasis maintenance of AKT/mTOR signaling

**Fig. 5:**
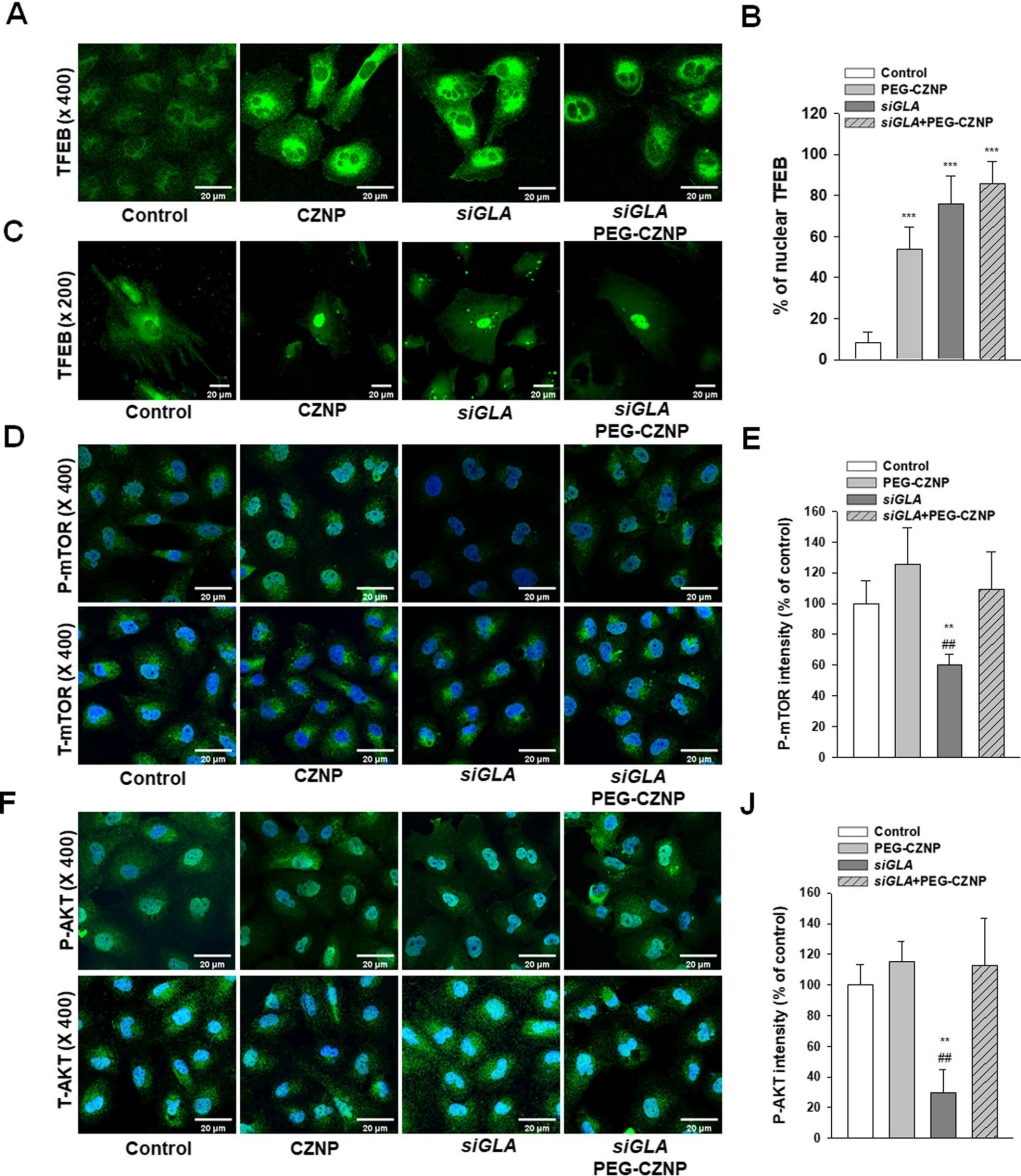
Effects of PEG-CZNPs on the TFEB and AKT/mTOR signaling pathways **A**, **B** Confocal microscopy images of HK-2 cells transfected with pEGFP-TFEB and quantification of the nuclear TFEB percentages. **C** Confocal microscopy images of human podocytes transfected with pEGFP-TFEB **D–J** HK-2 cell analysis **D**, **E** Confocal microscopy image of cells stained with P-mTOR and T-mTOR and quantification of the fluorescence intensities using ImageJ software. **F** Confocal microscopy images of cells stained with P-AKT and T-AKT and quantification of the fluorescence intensities using ImageJ software. Data values represent the mean ± SD; **P < 0.01 and ***P < 0.001 versus control; ^*##*^*P* < 0.01 versus s*iGLA* knockdown alone

To further investigate the impact of PEG-CZNPs on intracellular autophagy, we evaluated the AKT/mTOR pathway and TFEB nuclear translocation using IFA. The fraction of TFEB puncta co-localized with the nucleus was increased in PEG-CZNP treated cells compared to untreated cells, indicating increased autophagosome synthesis. The fluorescence intensity of p-AKT and p-mTOR was found to be significantly decreased in *GLA* knockdown HK-2 cells compared to the control HK-2 cells. Notably also, PEG-CZNP treatment significantly increased the p-AKT and p-mTOR levels. These data suggest that the increased TFEB nuclear translocation induced by PEG-CZNPs increases the AKT and mTOR activity levels in *GLA* knockdown HK-2 cells and it is likely that this helps to maintain the balance of the autophagy flux in the cellular models of FD (Fig. 5A–J).

### PEG-CZNPs attenuate the ROS levels in HK-2 cell and human podocyte models of FD

**Fig. 6:**
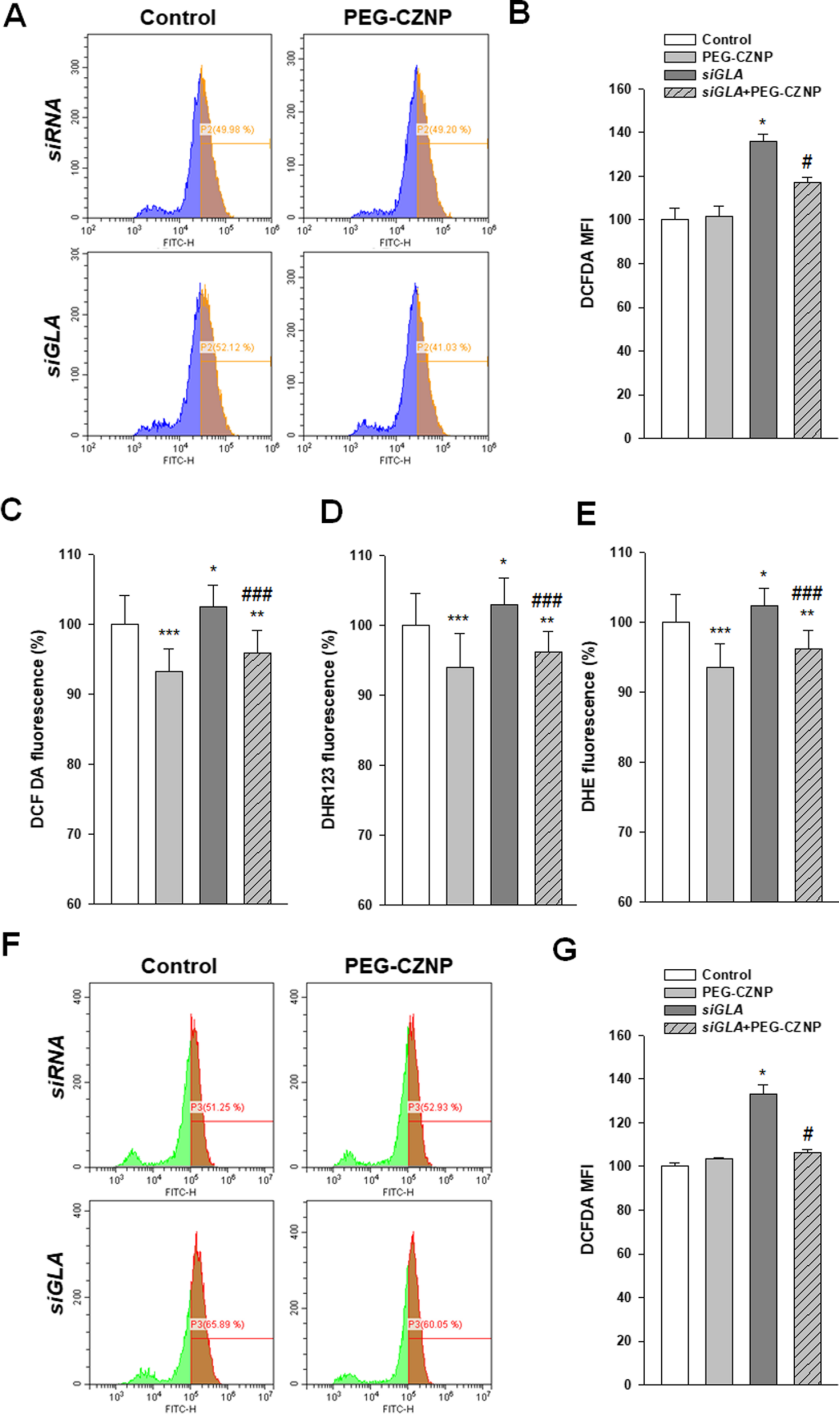
Effects of PEG-CZNPs on ROS production **A-E** HK-2 cell model of FD at day 8 **A, B** DCF-DA analysis in HK-2 cells using a FACS assay. **C-E** Intracellular ROS level measurements using DCF-DA, DHR and DHE with or without PEG-CZNP exposure. **F, G** DCF-DA analysis in human podocytes using a FACS assay. Data values represent the mean ± SD; *P < 0.05, *P < 0.01 and ***P < 0.001 versus control; ^#^P < 0.01 and ^###^P < 0.001 versus *siGLA* knockdown alone

We evaluated the changes to the ROS levels in HK-2 cells and human podocytes after a *GLA* knockdown, with or without PEG-CZNP treatment, using DHE staining, DCF-DA and DHR assays. We utilized fluorescence activated cell sorting (FACS) or a microplate fluorescence reader to generate the data in these analyses. The intracellular Gb3 accumulation caused by the *GLA* knockdown induced a significant elevation in ROS production, but this effect was significantly suppressed by PEG-CZNP treatment in both cell models of FD (Fig. 6A–G).

### PEG-CZNPs attenuate apoptosis in the HK2 cell model of FD

**Fig. 7:**
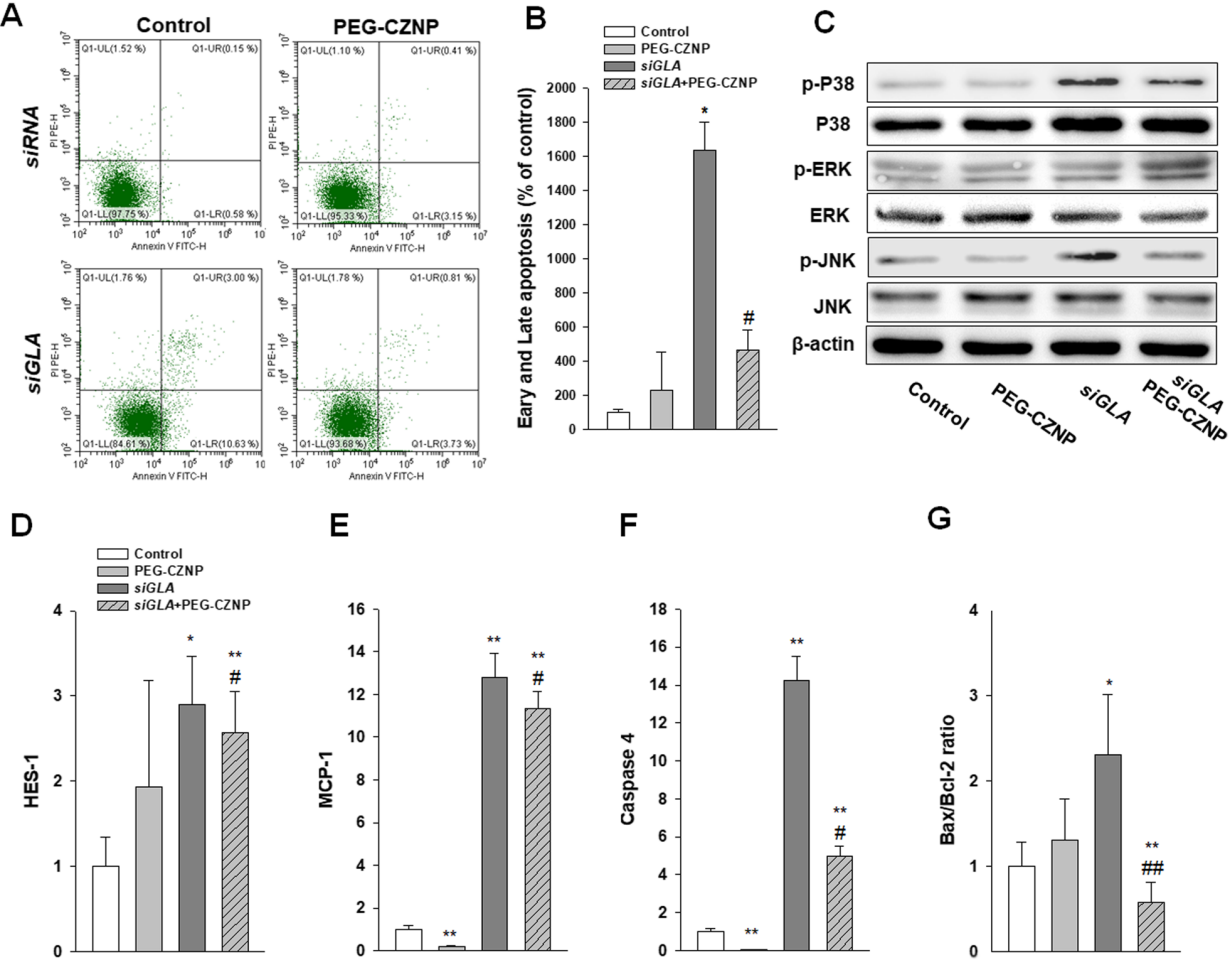
Beneficial effects of PEG-CZNPs on cellular survival and apoptosis in HK-2 cell model of FD. **A, B** Analysis of apoptosis in HK-2 cells using a FACS assay **C** Immunoblotting of MAPK dependent intracellular signaling. **D-F** Quantitative real-time RT-PCR analysis of HES-1, MCP-1, caspase 4, *Bax* and *Bcl-2* gene expression. Data values represent the mean ± SD; *P < 0.05, and **P < 0.01, versus control; ^#^P < 0.05, and ^##^P < 0.01 versus *siGLA* knockdown alone

The impact of PEG-CZNPs on Gb3 induced apoptosis was assessed via the FACS analysis of HK-2 cells using Annexin V-FITC conjugation. The apoptotic response was found to be significantly increased after the *GLA* knockdown but attenuated in these same cells by exposure to PEG-CZNPs. We validated these changes by measuring *Bax* and *Bcl-2* expression by real time RT-PCR. To further delineate the signaling mechanisms underlying Gb3 induced apoptosis, we analyzed the activation of the stress responsive mitogen-activated protein kinase (MAPK) pathways p38, JNK and ERK. Gb3 accumulation also led to increased phosphorylation of p38 and JNK, and this effect was again blocked by PEG-CZNP administrations (Fig. 7A–G).

### PEG-CZNPs suppress fibrosis marker expression in the human podocyte model of FD

**Fig. 8:**
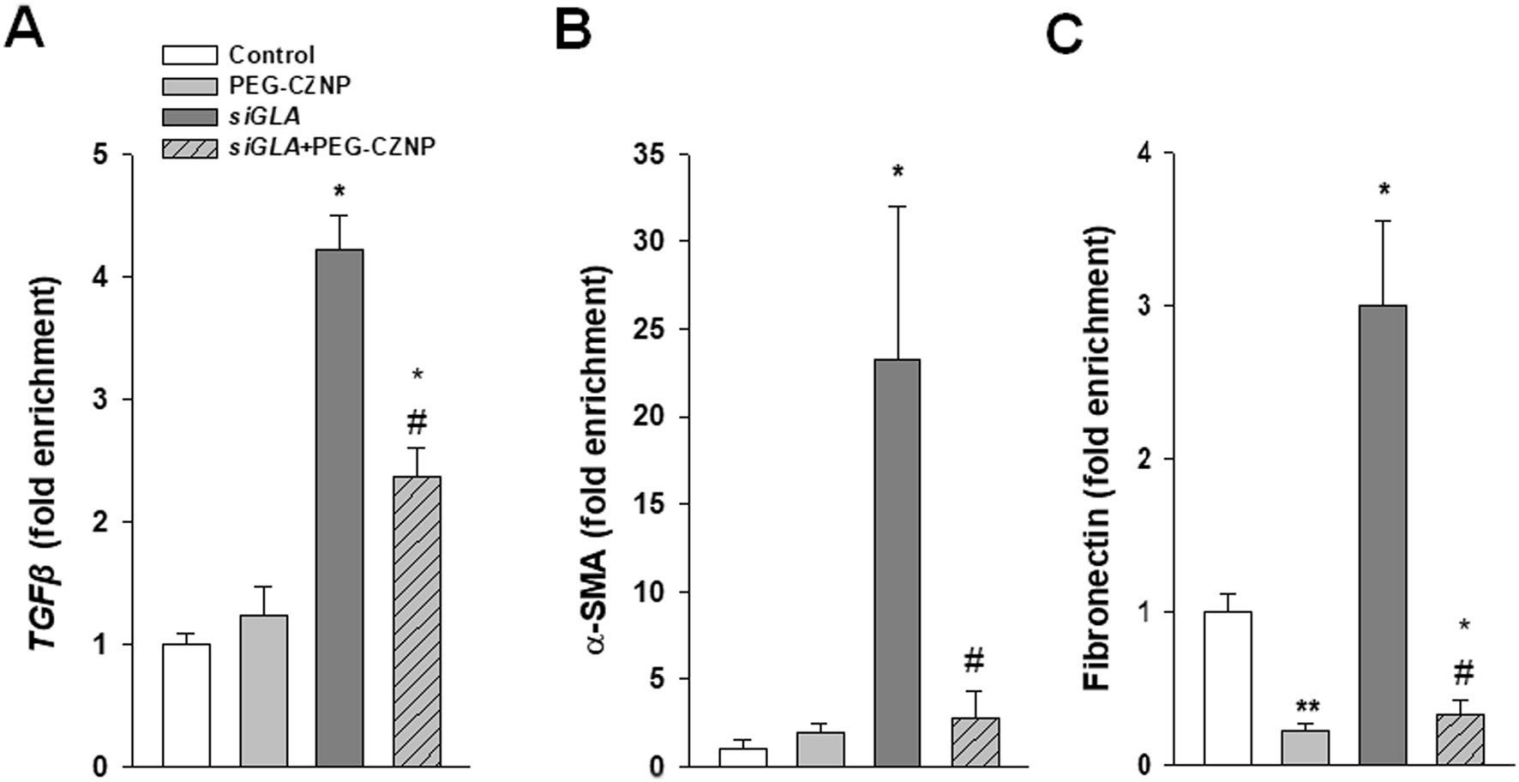
Effects of PEG-CZNPs on fibrosis markers in the human podocyte model of FD. **A–C** Quantitative real-time RT-PCR analysis of TGFß, α-SMA and fibronectin expression. Data values represent the mean ± SD; *P < 0.05, and **P < 0.01, versus control; ^#^P < 0.05, and ^##^P < 0.01 versus *siGLA* knockdown alone

We conducted RT-PCR analysis to investigate the effect of the PEG-CZNPs on fibrosis in the human podocyte model of FD. The expression of fibrosis markers such as *TGFβ1*, *fibronectin* and *α SMA* was found to be significantly increased in the *GLA* knockdown podocytes, an effect which was suppressed in these cells by PEG-CZNP exposure (Fig. 8A–C).

### PEG-CZNPs attenuate kidney tissue Gb3 accumulation and podocyte injury in ***B6;129-Gla***^***tm1Kul***^/J FD model mice

We next analyzed changes in kidney tissue Gb3 accumulation and kidney injury in 12-week –old male *B6;129-Gla*^*tm1Kul*^/J mice and age matched wild type B6 mice, with or without PEG-CZNP treatment. The results showed that α-Gal A activities in the serum of the *B6;129-Gla*^*tm1Kul*^/J FD model mice were significantly reduced compared to wild type animals (Additional file 1: Fig. S8). The serum BUN and creatinine levels were unchanged however between all of the study animal groups (Table 1).

**Table 1:** Renal function assessments in the mouse study groups (mean ± SD)

Parameter	Group 1 (n = 3)	Group 2 (n = 3)	Group 3 (n = 5)	Group 4 (n = 5)
BUN (mg/dL)	23.70 ± 3.90	20.97 ± 4.04	24.58 ± 4.05	24.36 ± 1.42
Cr (mg/dL)	0.24 ± 0.04	0.17 ± 0.04	0.21 ± 0.11	0.22 ± 0.06

The data values are the mean ± SD, *BUN* blood urea nitrogen, *Cr* creatinine. Group1; wild type, normal saline injection, Group 2; wild type + PEG-CZNP injection, Group 3; *B6;129-Gla*^*tm1Kul*^/J mice + normal saline injection and Group 4; *B6;129-Gla*^*tm1Kul*^/J mice + PEG-CZNP injection

**Fig. 9:**
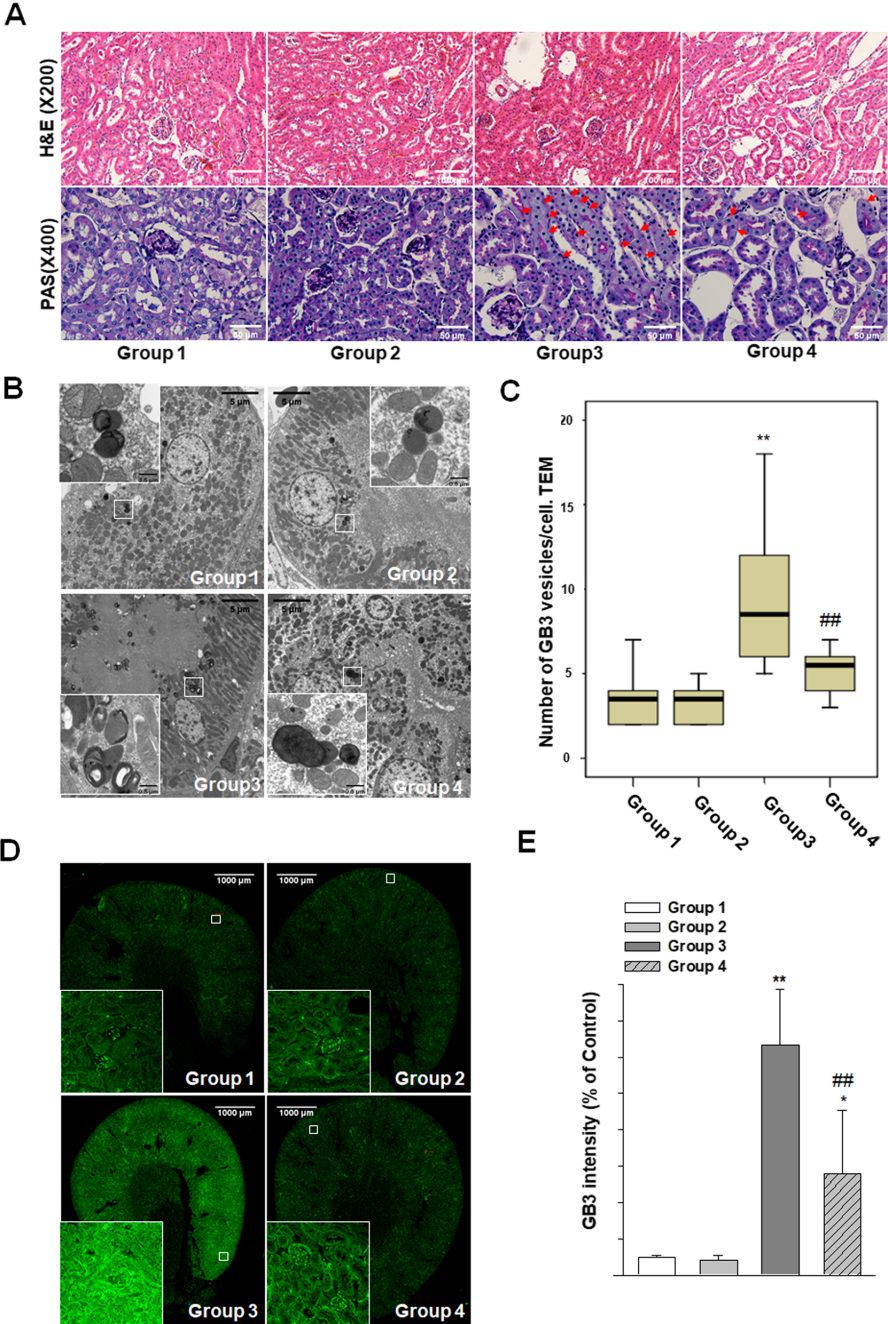
Renal histopathology analysis in a *B6;129-Gla*^*tm1Kul*^/J mouse model of FD, with or without PEG-CZNP exposure. **A** Representative photomicrographs are shown of H&E and PAS stained sections from kidney tissues isolated from the mice at 12 weeks of age, with or without PEG-CZNP pretreatment. Enlargement of the tubular cells was alleviated in the *B6;129-Gla*^*tm1Kul*^/J mice upon PEG-CZNP exposure (red arrow). **B**, **C** Representative TEM images and quantification of the number of Gb3 vesicles. **D**, **E** Confocal micrographs of Gb3-stained kidney sections and quantification of the Gb3 signal intensity using ImageJ software. Group1; wild type, normal saline injection, Group 2; wild type + PEG-CZNP injection, Group 3; *B6;129-Gla*^*tm1Kul*^/J mice + normal saline injection and Group 4; *B6;129-Gla*^*tm1Kul*^/J mice + PEG-CZNP injection. Data values represent the mean ± SD; **P < 0.01 versus Group1; ^##^P < 0.01 versus Group 3 alone

**Fig. 10:**
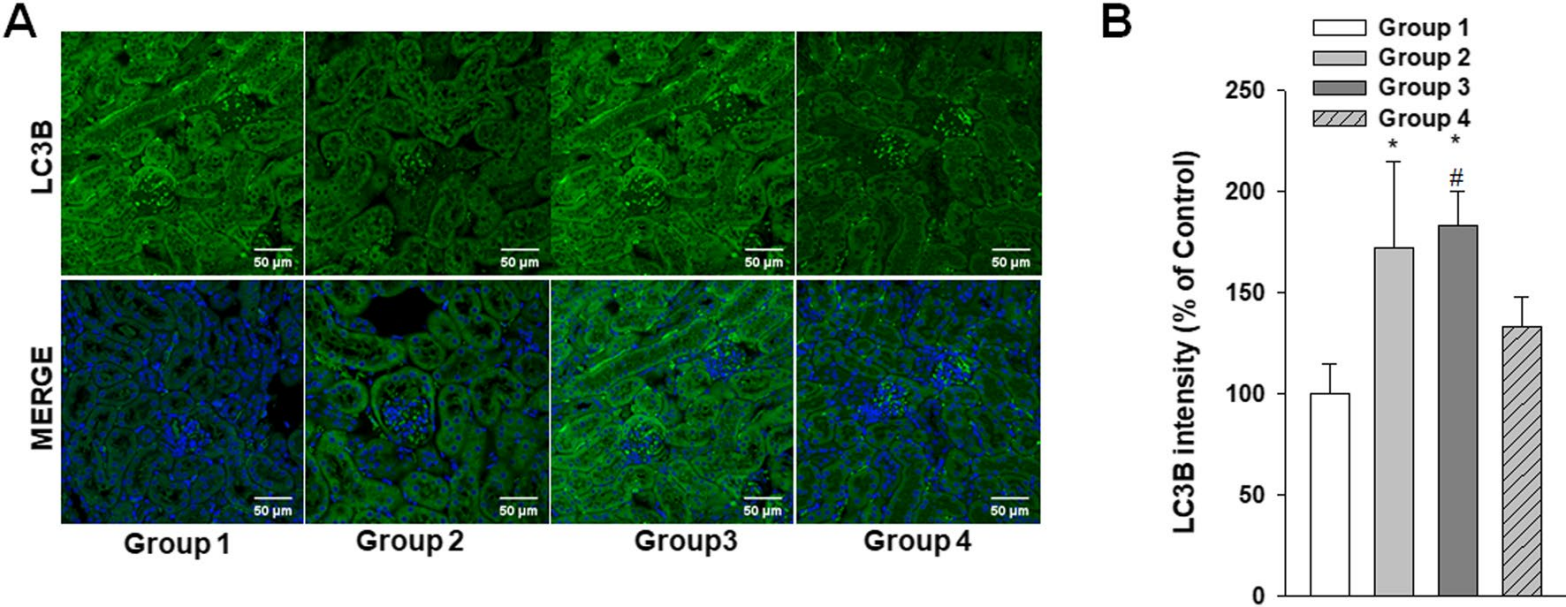
Effects of PEG-CZNPs on the autophagy response in *B6;129-Gla*^*tm1Kul*^/J mouse model of FD. **A** Representative confocal immunofluorescence microscopy images of LC3b (green), with DAPI counterstaining (blue), in control and α-GLA deficient *B6;129-Gla*^*tm1Kul*^/J mice with or without PEG-CZNP exposure. **B** Quantification of LC3B intensity using ImageJ software. Group1; wild type, normal saline injection, Group 2; wild type + PEG-CZNP injection, Group 3; *B6;129-Gla*^*tm1Kul*^/J mice + normal saline injection and Group 4; *B6;129-Gla*^*tm1Kul*^/J mice + PEG-CZNP injection. Data values represent the mean ± SD; *P < 0.05 versus Group 1; ^#^P < 0.05 versus Group 3 alone

**Fig. 11:**
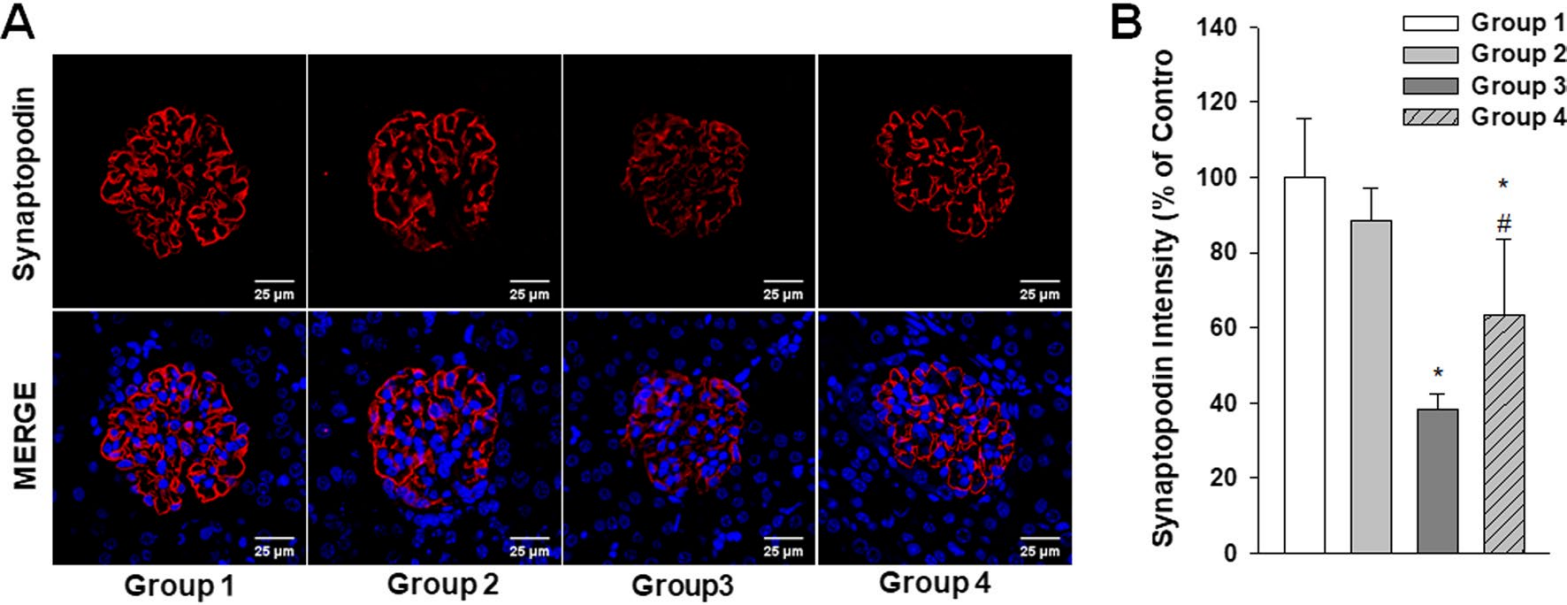
Effects of PEG-CZNPs on podocyte injury in the *B6;129-Gla*^*tm1Kul*^/J mouse model of FD. **A** Representative confocal immunofluorescence microscopy images of synaptopodin (red), with DAPI counterstaining (blue), in control and α-GLA deficient *B6;129-Gla*^*tm1Kul*^/J mice with or without PEG-CZNP exposure. **B** Quantification of the synaptopodin intensities using ImageJ software. Group1; wild type, normal saline injection, Group 2; wild type + PEG-CZNP injection, Group 3; *B6;129-Gla*^*tm1Kul*^/J mice + normal saline injection and Group 4; *B6;129-Gla*^*tm1Kul*^/J mice + PEG-CZNP injection. Data values represent the mean ± SD; *P < 0.05 versus Group 1; ^#^P < 0.05 versus Group 3 alone

Histologic changes were also analyzed in these experimental mice by H&E and PAS staining. Although enlarged tubular cells were observed in the *B6;129-Gla*^*tm1Kul*^/J FD model mouse kidneys, no structural abnormalities were evident (Fig. 9A). EM analysis revealed large lipid inclusions with electron-dense concentric lamellar structures in the kidney tubules from the *B6;129-Gla*^*tm1Kul*^/J mice and that PEG-CZNP treatments successfully suppressed these effects (Fig. 9B, C). The kidney tissues Gb3 levels were measured again in these animals by IF and the fluorescence intensity of the Gb3 staining was significantly increased in the *B6;129-Gla*^*tm1Kul*^/J mice but decreased after PEG-CZNP treatment (Fig. 9D, E). We further performed IFA with LC3B staining to investigate autophagy changes in these kidney tissues. The fluorescence intensity of LC3B was significantly increased in Group 3 but decreased in Group 4 (Fig. 10A, B). To analyze whether PEG-CZNPs would protect against podocyte injury in our mouse model of FD, we performed IF analysis of the kidney tissue with synaptopodin. Fluorescence intensity of this marker was significantly decreased in Group 3 but restored to normal levels by PEG-CZNP treatment (Fig. 11A, B).

**Fig. 12:**
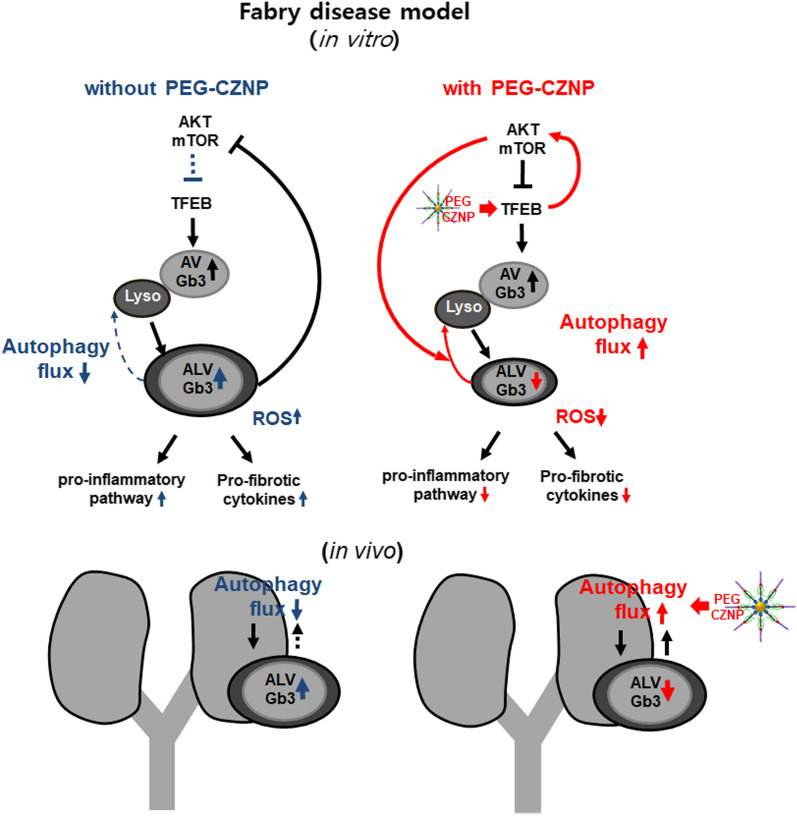
Schematic illustrations of the PEG-CZNP induced attenuation of kidney injury from FD. This occurs via the enhancement of the autophagy flux combined with the activation of TFEB, restoration of AKT/mTOR signaling, and antioxidant effects. PEG-CZNPs thereby alleviate inflammatory and fibrosis pathways and attenuate Gb3-mediated kidney injury

## Discussion

We show from our current analysis that intracellular Gb3 depositions combined with a disruption to the cellular autophagy flux, increases the intracellular oxidative stress, augments the inflammatory response, and promotes cell death and fibrosis in a cellular model of FD. These processes will ultimately lead to kidney tissue injury and it was significant, they were attenuated by PEG-CZNPs which restored a functional autophagy flux, and also had antioxidant effects. To the best of our knowledge, our present study is the first to demonstrate that the progression of FD can be attenuated by a nanoparticle and suggest that this is a potentially novel therapeutic autophagy regeneration agent (Fig. 12).

In accordance with previous studies [17–20], our current findings showed that the autophagy flux was significantly impaired in separate cellular models of FD. We confirmed that a dysfunctional autophagy flux was present in HK-2 cell and human podocyte models of FD when these cells were exposed to CQ, a known autophagy inhibitor which prevents the autophagosome from merging with lysosome via the inhibition of lysosomal acidification [42]. We observed an increased LC3BII/I ratio following CQ administration in the cells containing PEG-CZNPs. Notably in our current study, PEG-CZNP treatments successfully decreased the intracellular Gb3 accumulation levels in both in vitro and in vivo models of FD by enhancing the autophagy flux.

The mechanism of autophagy dysfunction in FD has been reported to involve the ATK/mTOR signaling pathway which is inhibited by intracellular Gb3 accumulation [18]. Overactive autophagy was thus thought to be a contributor to podocyte damage in FD. mTOR has been reported to not only deactivate autophagy but also contribute to regeneration of autophagosomes and lysosomes from autolysomal vesicles [43]. Interestingly, deactivated AKT and mTOR signaling pathway in HK-2 cell model of FD recovered by PEG-CZNP treatments. It known that CNP promotes autophagy by activating TFEB [28]. In accordance with the findings of a previous study, PEG-CZNP exposure in our current FD models also enhanced TFEB nuclear translocation. TFEB was reported previously to participate in a negative feedback loop with mTOR1 by directly inducing the expression of RRAFD and thus enhancing mTORC1 activity [44, 45]. TFEB activation was also reported to activate AKT signaling via the upregulation of insulin receptor substrate (IRS) [46]. We thus speculated that the activation of TFEB by PEG-CZNPs operates via a negative feedback process with the AKT/mTOR pathway, and that this may be the mechanism by which these particles attenuate overactive autophagy and reduce cellular damage.

Gb3 accumulation has been demonstrated previously to induce ROS production, and mitochondrial SOD2 suppression has been reported as one of the causes of vascular endothelial dysfunction in FD [47–50]. Although the pathways by which elevated intracellular Gb3 induces ROS production are still unclear, it is thought that this involves the activation of oxidative enzymes. In accordance with the results of previous studies, ROS levels were increased in both the HK-2 cell and podocyte models of FD used in our current study but attenuated by PEG-CZNP exposure of these cells. The mechanism of ROS reduction after PEG-CZNP treatment may be due to the decreases Gb3 accumulation and intrinsic antioxidant effects of the PEG-CZNPs.

We further found in our current study that the JNK and p38 pathways play a role in mediating Gb3 accumulation-induced HK-2 cell apoptosis. The phosphorylation of JNK and p38 was also found to be decreased, and that of ERK increased, by PEG-CZNP administration in our HK-2 cell model of FD. Our results were thus consistent with the findings in previous studies that p38 and JNK are involved in apoptosis and ERK is involved in cellular survival in FD [51, 52].

TGFβ, α-SMA and fibronectin, which are well-established fibrosis markers were significantly increased in the podocyte model of FD in our current study. Podocytes are the earliest cells to be loaded with glycolipid deposits and Gb3 has been proposed as a promoter of fibrosis in FD [53]. Hence, Gb3 increases both the expression of the fibrogenic cytokine TGFβ1 and the synthesis of extra cellular matrix (ECM) components such as fibronectin and type IV collagen in a TGFβ1 dependent manner in podocytes [54–56]. Notably, these fibrosis markers were observed to be decreased after PEG-CZNP treatment in the hunam podocyte model of FD in our current study. Moreover, in in vivo FD mouse model, one of the podocyte–specific markers, synaptopodin, was found to be still conserved in the PEG-CZNP treatment group. We hypothesized from this that PEG-CZNPs contribute to the maintenance of podocyte characteristics in FD and will have protective effects against Gb3 induced fibrosis. In our current animal study, the observation time of three months was relatively short for fibrosis to appear, so a longer period of observation is needed in the future study to more clearly confirm the effect of PEG-CZNP on fibrosis.

In our present study, no significant toxicity was observed in mice after administration of 10 mg/kg of PEG-CZNPs via intraperitoneal injection twice a week for 12 weeks. PEG-CZNPs mostly accumulated in spleen and liver with a minor fraction identified in the lung and kidneys, and undetectable in brain. These are in accordance with previous studies [32, 57]. The deposition of CNPs in spleen and liver is thought to be due to the removal of NPs by reticuloendothelial system and immune macrophage/kupffer cells [58].

In summary, the results of our current study indicated the successful uptake of PEG-CZNPs into kidney cells and tissues where they effectively lowered Gb3 accumulation by enhancing the autophagy flux and TFEB activation, blocked the progression of apoptosis and fibrosis, and promoted ROS scavenging. These findings provide the first demonstration that PEG-CZNPs alleviate the progression of kidney injury in FD and that these particles may form the basis for developing novel treatments for this disorder.

## Supplementary Information

**Additional file 1: Fig S1**. Schematic representation of synthesized PEG-CZNPs and FITC conjugated PEG-CZNPs. A The process comprises two steps; (i) preparation of CZNPs based on non-hydrolytic sol-gel reaction in oleylamine; (ii) transfer of the as-synthesized CZNPs to aqueous phase by modifying with phospholipid-PEG. B FITC-conjugated phospholipid-PEG capped CZNPs were prepared with a thiourea linkage. C Analysis of Fourier transform infrared spectroscopy (FT-IR) spectra comparing the as synthesized PEG-CZNP and FITC conjugated PEG-CZNP with FITC. (a) PEG-CZNP, (b) FITC and (c) FITC conjugated PEG-CZNP. **Fig. S2**. Expression of key differentiation markers in human podocytes. A, B Representative confocal immunofluorescence microscopy images of podocin, nephrin and synaptopodin (green), with DAPI counterstaining (blue), in both undifferentiated and differentiated human podocytes. **Fig. S3**. Validation of α-GLA knockdown efficiency by quantitative real-time RT-PCR analysis and immunoblotting. A, B Quantitative real-time RT-PCR and immunoblotting results showing the α-GLA knockdown efficiency in HK-2 cells and human podocytes. Data values represent the mean ± SD; *P<0.05 and **P<0.01 versus control. **Fig. S4**. Effects of PEG-CZNPs on cell viability. The viability of HK-2 cells (A) and matured human podocytes (B) treated with PEG-CZNP was measured using the MTT assay. Data values represent the mean ± SD; *P<0.05 and ***P<0.001 versus 0 μg/mL. **Fig. S5**. Schema for the PET-CZNP treatments of the mouse model of FD. PEG-CZNPs (10 mg/kg) or normal saline (2 mL/kg) were administered intraperitoneally to the mice twice per week from 4 to 12 weeks of age. The mice were sacrificed at 12 weeks of age. **Fig. S6**. Intracellular localization and biodistribution of the PEG-CZNPs. Confocal microscopy analysis of human podocyte treated with FITC-labeled PEG-CZNPs (green) after 0, 4, 8, 16, 24, 48, 72 and 96 hr. The human podocytes were then stained with Lysotracker (red). **Fig. S7**. Biodistribution analysis of PEG-CZNPs. ICP-MS analysis of the ceria (A) and zirconia (B) contents in the organs of B6 mice after the intraperitoneal injection of 10mg/kg PEG-CZNPs twice a week for 12 weeks. **Fig. S8**. ELISA measurements of the GLA protein levels in plasma samples from the mouse model of FD. Group1; wild type, normal saline injection, Group 2; wild type + PEG-CZNP injection, Group 3; B6;129-Gla^tm1Kul^/J mice + normal saline injection and Group 4; B6;129-Gla^tm1Kul^/J mice + PEG-CZNP injection. Data values represent the mean ± SD; *P<0.05, ***P < 0.001 versus Group 1.

## Data Availability

All data are fully available without restriction.

## Abbreviations

FD: Fabry disease
LSD: Lysosome storage disease
PEG-CZNP: Polyethyleneglycol-capped Ceria-Zirconia antioxidant nanoparticles
Gb3: Globotriaosylceramide
HK-2: Human renal proximal tubular epithelial cells
α-GLA: α Galactosidase A
ROS: Reactive oxygen species
ERT: Enzyme replacement therapy
NPs: Nanoparticles
CNPs: Ceria nanoparticles
FITC: Fluorescein isothiocyanate
DW: Deionized water
PEG: Polyethylene glycol
TEM: Transmission electron microscopy
XRD: Multi-purpose X ray-diffractometer
SAED: Selected area electron diffraction
STEM: Scanning transmission electron microscope
TGA: Thermogravimetric analysis
DLS: Dynamic light scattering measurements
EDS: Energy-dispersive X-ray spectroscopy
DPPH: 1,1-Diphenyl-2-picrylhydrazyl
FBS: Fetal bovine serum
siRNA: Small interfering RNA
TFEB: Transcription factor EB
MTT: 3-(4,5-Dimethylthiazole-2-yl)-2,5-diphenyltetrazolium bromide
hr: Hours
RIPA: Radioimmunoprecipitation assay
SDS-PAGE: Sodium dodecyl sulfate–polyacrylamide gel electrophoresis
PVDF: Polyvinylidene difluoride
TBS: Tris-buffered saline
p-JNK: Phpospho- c-Jun N-terminal kinase
LC3B: Microtubule-associated proteins 1A/1B light chain 3B
ERK: Extracellular signal-regulated kinase
DHE: Dihydroethidium
DCF-DA: Dichlorodihydrofluorescein diacetate
DHR: Dihydrorhodamine
p-mTOR: Phospho- mammalian target of rapamycin
p-AKT: Phospho-protein kinase B
PBS: Phosphate-buffered saline
PFA: Paraformaldehyde
DAPI: Diamidino-2-phenylindole
LC–ESI–MS/MS: Liquid Chromatography coupled to Electrospray Ionization Tandem Mass Spectrometry
MRM: Multiple Reaction Monitoring
PI: Propidium iodide
BUN: Blood urea nitrogen
H&E: Hematoxylin–eosin
PAS: Periodic Acid Schiff
IFA: Immunofluorescence assay
CQ: Chloroquine,
FACS: Fluorescence activated cell sorting
MAPK: Mitogen-activated protein kinase
JNK: C-Jun-N-terminal kinase
WT: Wild type
EM: Electron microscopy
SOD2: Superoxide dismutase 2
SMA: Smooth muscle chain
ECM: Extra cellular matrix
