# Supplementary material for: Ceria-Zirconia nanoparticles reduce intracellular globotriaosylceramide accumulation and attenuate kidney injury by enhancing the autophagy flux in cellular and animal models of Fabry disease
Source: J Nanobiotechnology. 2022 Mar 9;20:125. doi: 10.1186/s12951-022-01318-8 (PMC8905732; doi:10.1186/s12951-022-01318-8)
Supplement: Supplementary file 1 — Additional file 1: Fig S1. Schematic representation of synthesized PEG-CZNPs and FITC conjugated PEG-CZNPs. A The process comprises two steps; (i) preparation of CZNPs based on non-hydrolytic sol-gel reaction in oleylamine; (ii) transfer of the as-synthesized CZNPs to aqueous phase by modifying with phospholipid-PEG. B FITC-conjugated phospholipid-PEG capped CZNPs were prepared with a thiourea linkage. C Analysis of Fourier transform infrared spectroscopy (FT-IR) spectra comparing the as synthesized PEG-CZNP and FITC conjugated PEG-CZNP with FITC. (a) PEG-CZNP, (b) FITC and (c) FITC conjugated PEG-CZNP. Fig. S2. Expression of key differentiation markers in human podocytes. A, B Representative confocal immunofluorescence microscopy images of podocin, nephrin and synaptopodin (green), with DAPI counterstaining (blue), in both undifferentiated and differentiated human podocytes. Fig. S3. Validation of α-GLA knockdown efficiency by quantitative real-time RT-PCR analysis and immunoblotting. A, B Quantitative real-time RT-PCR and immunoblotting results showing the α-GLA knockdown efficiency in HK-2 cells and human podocytes. Data values represent the mean ± SD; *P<0.05 and **P<0.01 versus control. Fig. S4. Effects of PEG-CZNPs on cell viability. The viability of HK-2 cells (A) and matured human podocytes (B) treated with PEG-CZNP was measured using the MTT assay. Data values represent the mean ± SD; *P<0.05 and ***P<0.001 versus 0 μg/mL. Fig. S5. Schema for the PET-CZNP treatments of the mouse model of FD. PEG-CZNPs (10 mg/kg) or normal saline (2 mL/kg) were administered intraperitoneally to the mice twice per week from 4 to 12 weeks of age. The mice were sacrificed at 12 weeks of age. Fig. S6. Intracellular localization and biodistribution of the PEG-CZNPs. Confocal microscopy analysis of human podocyte treated with FITC-labeled PEG-CZNPs (green) after 0, 4, 8, 16, 24, 48, 72 and 96 hr. The human podocytes were then stained with Lysotracker (red). Fig. S7. Biodistributio [file 12951_2022_1318_MOESM1_ESM.docx]

***Supplementary methods, figures and references for:***

**Ceria-zirconia nanoparticles reduce intracellular globotriaosylceramide accumulation and attenuate kidney injury by enhancing the autophagy flux in cellular and animal models of Fabry disease**

Jong Hun An^1^**^†^**, Sang-Eun Hong^2^**^†^**, Seong-Lan Yu^3^, Jaeku Kang^4^, Chang Gyo Park^4^, Hoi Young Lee^4^, Sung-Ki Lee^3,5^, Dong Chul Lee^6^, Hwan-Woo Park^7^, Won-Min Hwang^1^, Sung-Ro Yun^1^, Yohan Park^1^, Moon Hyang Park^8^, Kuk Ro Yoon^2^, Se-Hee Yoon^1*^

**^†^**Both authors contributed equally to this work.

^*^Corresponding author: [sehei@hanmail.net](mailto:sehei@hanmail.net)

^1^Division of Nephrology and Department of Internal Medicine, Myunggok Medical Research Institute, College of Medicine, Konyang University, Daejeon, Republic of Korea,

^2^Department of Chemistry, Hannam University, Daejeon, Republic of Korea

^3^Myunggok Medical Research Institute, College of Medicine, Konyang University, Daejeon, Republic of Korea,

^4^Department of Pharmacology, College of Medicine, Konyang University, Daejeon, Republic of Korea,

^5^Department of Obstetrics and Gynecology, College of Medicine, Konyang University, Daejeon, Republic of Korea,

^6^Personalized Genomic Medicine Research Center, Korea Research Institute of Bioscience and Biotechnology (KRIBB), Deajeon, Republic of Korea

^7^Department of Cell Biology, Myunggok Medical Research Institute, Konyang University College of Medicine, Daejeon, Republic of Korea

^8^Department of Pathology, College of Medicine, Konyang University, Daejeon, Republic of Korea

Supplemental methods

**Synthesis of PEG-CZNPs**

CZNPs were synthesized using a non-hydrolytic sol-gel reaction method as described previously [1]. Ce_0.7_Zr_0.3_O_2_ (7CZ) NPs are reported to have the highest superoxide dismutase mimetic effect among the various Ce_x_Zr_1−x_O_2_ particles [2]. Thus 7CZNPs were produced for our current study. Briefly, a 0.5 g mixture of cerium (III) acetylacetonate hydrate and zirconium (IV) acetylacetonate hydrate at an appropriate molar ratio was added to 15 mL of oleylamine. The mixture was then sonicated for 15 min at 20℃ and then heated to 80℃ at a rate of 2 ℃/min. The reaction mixture was aged at 80 ℃ for 1 day to obtain a dark brownish colloidal solution and then cooled to room temperature. Acetone (100 mL) was added to wash and precipitate the CZNPs by centrifugation, and the resulting particles were dispersed in chloroform to yield a final concentration of 10 mg/mL. For improving water dispensability, the CZNPs were encapsulated by a phospholipid polyethylene glycol (PEG) shell. Briefly, 5 mL of the NP sample in chloroform (10 mg/mL) was mixed with 10 mL of mPEG (2000)-PE in chloroform (10 mg/mL). The majority of the chloroform was then removed in a rotary evaporator and the sample was incubated at 70 ℃ for 2 h in a vacuum oven to ensure complete removal of this solvent. Next, 5 mL of deionized water was added to the sample to obtain a transparent colloidal suspension. After filtration using a 0.4 μM filter, the excess mPEG (2000)-PE was removed by ultracentrifugation. The purified phospholipid-PEG-capped sample was finally dispersed in DW.

**FITC conjugation of CZNPs**

To attach FITC to the NP surface, the CZNP samples were encapsulated using an amine-functionalized phospholipid-PEG shell as previously described [1]. Briefly, 5 mL of the NP sample in chloroform (10mg/mL) was mixed with 9 mL of mPEG (2000)-PE in chloroform (10 mg/mL) and 1mL of DSPD-PEG (2000) amine in chloroform (10 mg/mL). The purification steps used to prepare phospholipid-PEG capped CZNP were used to obtain NPs dispersed in DW. Then, a FITC-conjugated phospholipid-PEG capped CZNP was prepared with a thiourea linkage formed between the isothiocyanate group (R-N=C-S) of FITC and the amino group (-NH_2_) on amin-functionalized group of phospholipid-PEG shell (Additional file 1: Fig.S1B). Briefly, 5mg of FITC was added to the NP suspension, and the mixture was stirred for 12 hr at 40 ℃. After filtration and ultracentrifugation, the FITC ester-conjugated NPs were dispersed in DW. Fourier transform infrared spectroscopy (FT-IR) spectra of FITC-conjugated CZNPs didn’t show a characteristic absorption peak at 2035 cm^-1^, which corresponds to the isothiocyanate group. This suggested that the reaction of thiourea with isothiocyanate group of FITC and the amino group of phospholipid-PEG capped CZNPs has been successfully performed.

**Cell viability assay**

The 3-(4,5-dimethylthiazole-2-yl)-2,5-diphenyltetrazolium bromide (MTT) assay was used to determine the viability of HK-2 cells and mature podocytes, as described previously [1, 3-5]. Briefly, HK-2 cells were seeded in 96-well plates at a density of 3 × 10^5^ and mature podocytes were also seeded in 12-well plate at a density of 2 × 10^4^ cells/well. Both cells were cultured with various concentrations of PEG-CZNPs in a 96-well plate and incubated for 24 h at 37 ℃ under 5% CO_2_. After this incubation, a 5 mg/mL MTT solution (Sigma) was added to the wells. The supernatant was removed after a further 4 hr and 1 mL of dimethyl sulfoxide (DMSO) was then added to each well. Immediately after purple formazan crystals formed and dissolved, the solution was collected and pipetted into a 96-well plate. The optical density was then measured using a 590 nm microplate reader (Synergy HTX; BioTek Instruments, Inc.). Both cells were treated with PEG-CZNPs at different concentrations (0, 5, 10, 20, and 40 μg/mL) for 24 hrs.

**ICP-MS analysis for biodistribution of PEG-CZNP**

The total zirconia and ceria content was measured using ICP-MS in two B6 mice that were administered intraperitoneally with PEG-CZNPs twice a week for 12 weeks as described previously [1]. The extracted kidney was lyophilized in a freeze-dryer (Ilshin Biobase, Ede, Netherlands, TFD5503) for 48 hr. Tissue samples were digested with mixture of 5 mL of nitric acid (Sigma–Aldrich, St. Louis, MO) and 1 mL of hydrofluoric acid (Sigma–Aldrich) in 60 mL Teflon Digestion Vessel (150 ℃, overnight). After cooling down, 3 mL of nitric acid and 0.5 mL of hydrofluoric acid were added for a further overnight incubation at 150 ℃. The lid of vessel was then opened and heated to dryness, and 10 g of 1% nitric acid solution was added to measure the ICP-MS.

**Supplementary Figures**

**Supplementary Fig 1.** Schematic representation of synthesized PEG-CZNPs and FITC conjugated PEG-CZNPs. **A** The process comprises two steps; (i) preparation of CZNPs based on non-hydrolytic sol-gel reaction in oleylamine; (ii) transfer of the as-synthesized CZNPs to aqueous phase by modifying with phospholipid-PEG. **B** FITC-conjugated phospholipid-PEG capped CZNPs were prepared with a thiourea linkage. **C** Analysis of Fourier transform infrared spectroscopy (FT-IR) spectra comparing the as synthesized PEG-CZNP and FITC conjugated PEG-CZNP with FITC. (a) PEG-CPNP, (b) FITC and (c) FITC conjugated PEG-CZNP.


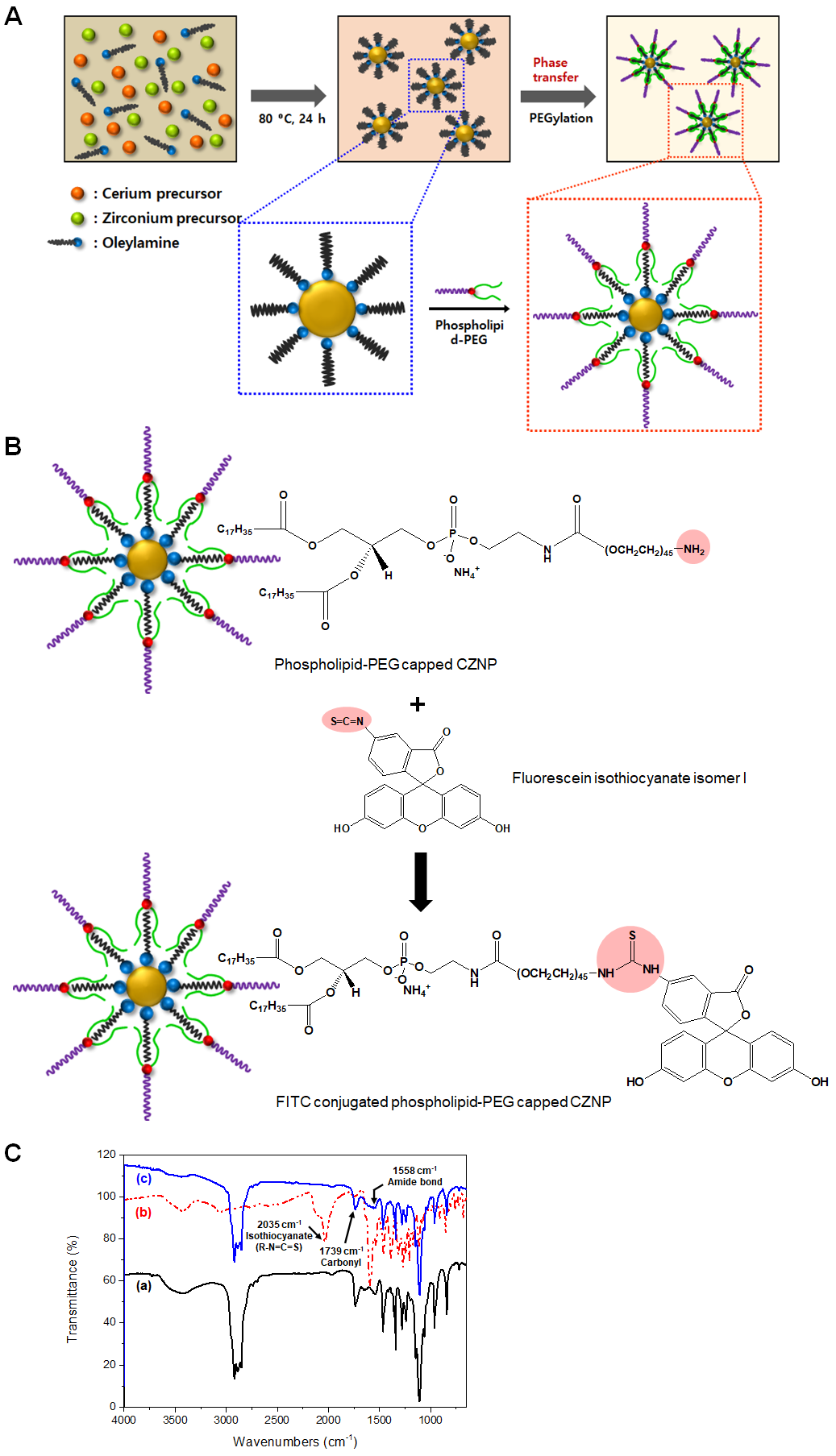


**Supplementary Fig. 2.** Expression of key differentiation markers in human podocytes. **A, B** Representative confocal immunofluorescence microscopy images of podocin, nephrin and synaptopodin (green), with DAPI counterstaining (blue), in both undifferentiated and differentiated human podocytes.


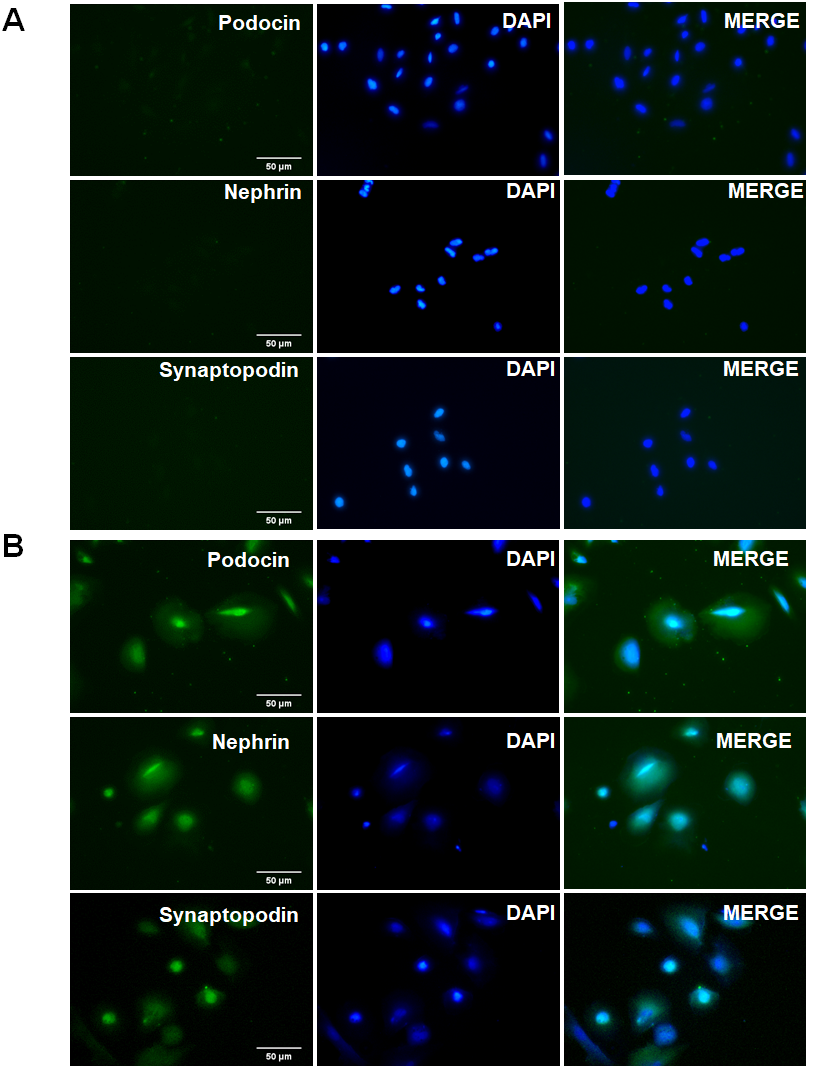


**Supplementary Fig. 3.** Validation of α-GLA knockdown efficiency by quantitative real-time RT-PCR analysis and immunoblotting. **A, B** Quantitative real-time RT-PCR and immunoblotting results showing the α-GLA knockdown efficiency in HK-2 cells and human podocytes. Data values represent the mean ± SD; *P<0.05 and **P<0.01 versus control.


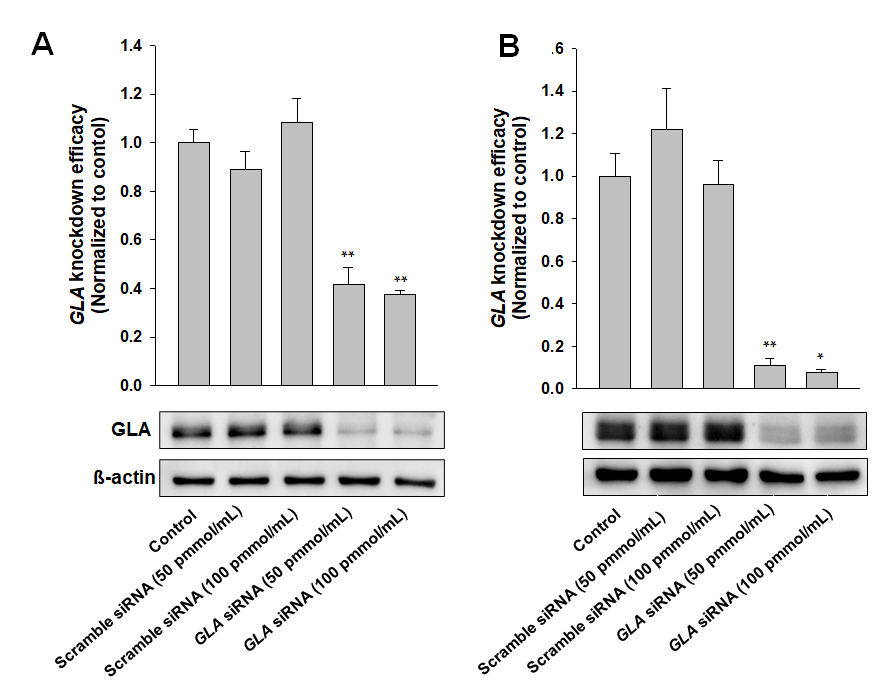


**Supplementary Fig. 4.** Effects of PEG-CZNPs on cell viability. The viability of HK-2 cells (A) and matured human podocytes (B) treated with PEG-CZNP was measured using the MTT assay. Data values represent the mean ± SD; *P<0.05 and ***P<0.001 versus 0 μg/mL.


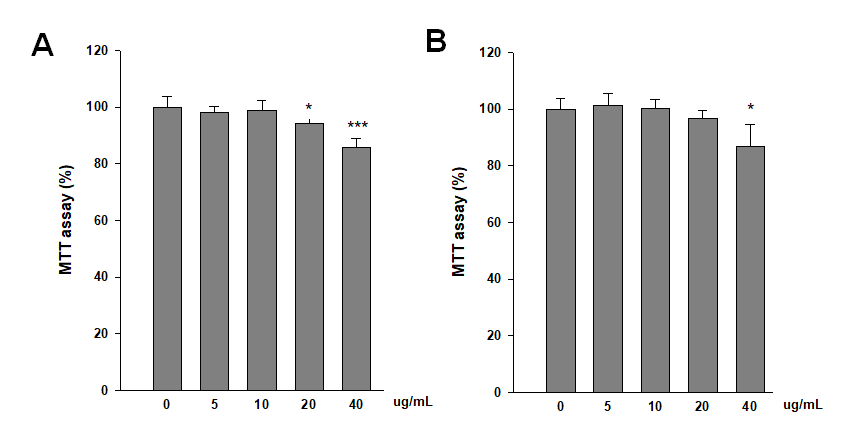


**Supplementary Fig. 5.** Schema for the PET-CZNP treatments of the mouse model of FD. PEG-CZNPs (10 mg/kg) or normal saline (2 mL/kg) were administered intraperitoneally to the mice twice per week from 4 to 12 weeks of age. The mice were sacrificed at 12 weeks of age.


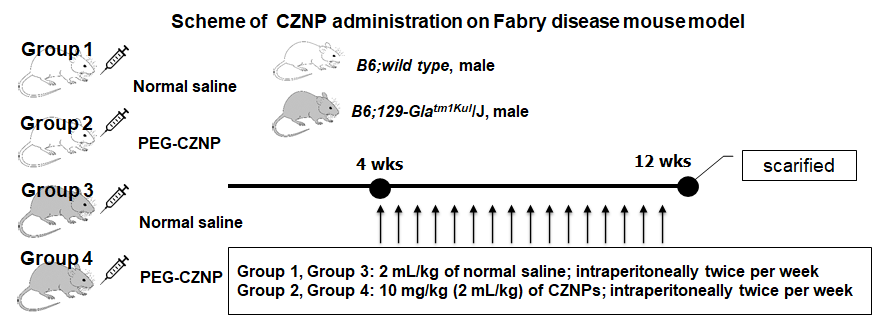


**Supplementary Fig. 6.** Intracellular localization and biodistribution of the PEG-CZNPs. Confocal microscopy analysis of human podocyte treated with FITC-labeled PEG-CZNPs (green) after 0, 4, 8, 16, 24, 48, 72 and 96 hr. The human podocytes were then stained with Lysotracker (red).


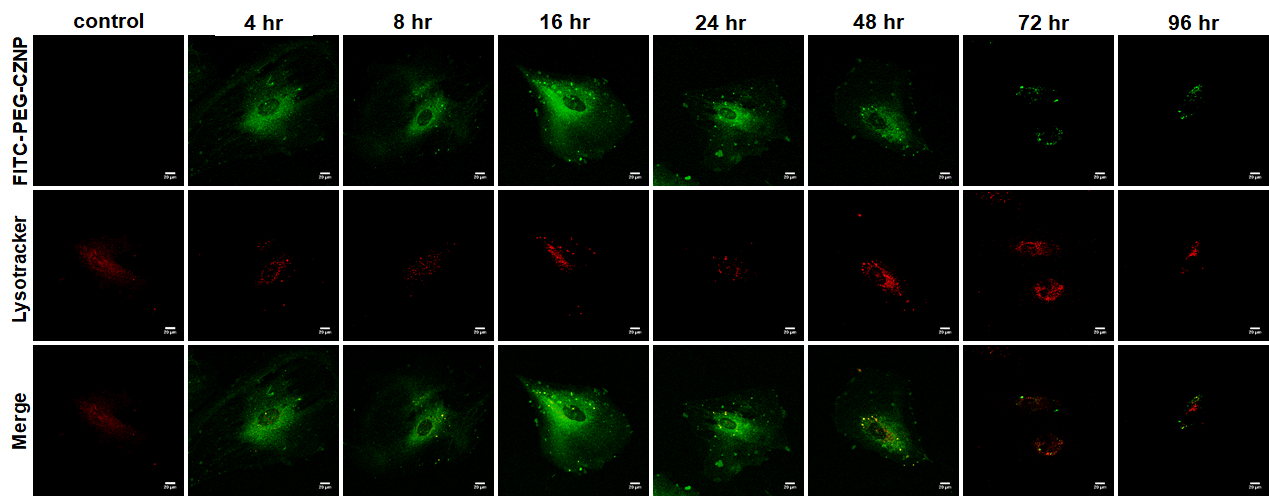


**Supplementary Fig. 7. Biodistribution analysis of PEG-CZNPs.** ICP-MS analysis of the ceria **(A)** and zirconia **(B)** contents in the organs of B6 mice after the intraperitoneal injection of 10mg/kg PEG-CZNPs twice a week for 12 weeks.


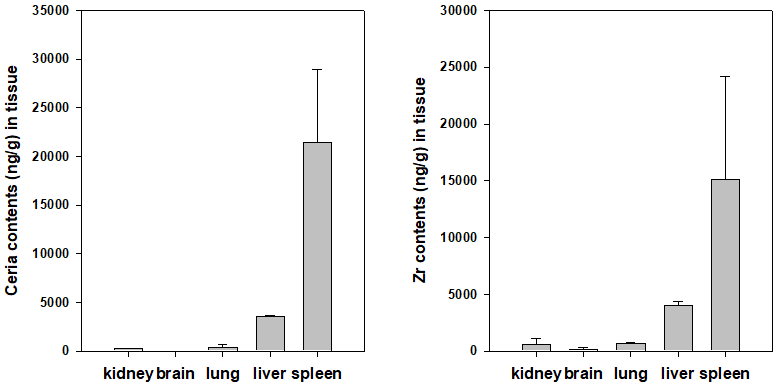


**Supplementary Fig. 8.** ELISA measurements of the GLA protein levels in plasma samples from the mouse model of FD. Group1; wild type, normal saline injection, Group 2; wild type + PEG-CZNP injection, Group 3; *B6;129-Gla^tm1Kul^*/J mice + normal saline injection and Group 4; *B6;129-Gla^tm1Kul^*/J mice + PEG-CZNP injection. Data values represent the mean ± SD; *P<0.05, ***P < 0.001 versus Group 1.


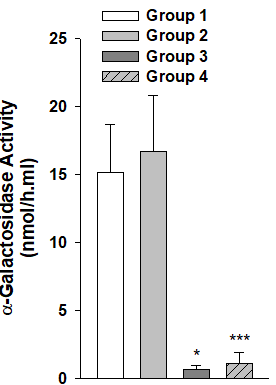


**Supplementary References**

1. Hong SE, An JH, Yu SL, Kang J, Park CG, Lee HY, et al. Ceria-Zirconia Antioxidant Nanoparticles Attenuate Hypoxia-Induced Acute Kidney Injury by Restoring Autophagy Flux and Alleviating Mitochondrial Damage. J Biomed Nanotechnol. 2020;16(7):1144-59.

2. Soh M, Kang DW, Jeong HG, Kim D, Kim DY, Yang W, et al. Ceria-Zirconia Nanoparticles as an Enhanced Multi-Antioxidant for Sepsis Treatment. Angew Chem Int Ed Engl. 2017;56(38):11399-403.

3. Cho S, Yu SL, Kang J, Jeong BY, Lee HY, Park CG, et al. NADPH oxidase 4 mediates TGF-beta1/Smad signaling pathway induced acute kidney injury in hypoxia. PLoS One. 2019;14(7):e0219483.

4. Jeong BY, Park SR, Cho S, Yu SL, Lee HY, Park CG, et al. TGF-beta-mediated NADPH oxidase 4-dependent oxidative stress promotes colistin-induced acute kidney injury. J Antimicrob Chemother. 2018;73(4):962-72.

5. Jeong BY, Lee HY, Park CG, Kang J, Yu SL, Choi DR, et al. Oxidative stress caused by activation of NADPH oxidase 4 promotes contrast-induced acute kidney injury. PLoS One. 2018;13(1):e0191034.
